# From kidney injury to cardiac dysfunction: the central role of oxidative stress in diabetes and CKD

**DOI:** 10.1007/s00395-025-01153-6

**Published:** 2025-12-19

**Authors:** Payel Sen, Theresa Sittig, Jules Hamers, Laura d’Ambrosio, Irem Ornek, Junqing Zhang, Bachuki Shashikadze, Jan B. Stöckl, Marie Bachter, Susanne Bierschenk, Simone Renner, Eckhard Wolf, Sebastian Clauss, Thomas Fröhlich, Alexander G. Nickel, Christoph Maack, Daphne Merkus

**Affiliations:** 1 Institute for Surgical Research, Walter Brendel Center of Experimental Medicine, University Clinic Munich, LMU Munich, Marchioninistrasse 68, 81377 Munich, Germany; 2 German Center for Cardiovascular Research (DZHK), Munich Heart Alliance (MHA), Partner Site Munich, Munich, Germany; 3 Interfaculty Center for Endocrine and Cardiovascular Disease Network Modelling and Clinical Transfer (ICONLMU), LMU Munich, Munich, Germany; 4 Gene Center, LMU Munich, Munich, Germany; 5 Center for Innovative Medical Models (CiMM), LMU Munich, Munich, Germany; 6 German Center for Diabetes Research (DZD), Neuherberg, Germany; 7 Department of Medicine I, University Hospital, LMU Munich, Marchioninistrasse 15, 81377 Munich, Germany; 8 Department of Translational Research, Comprehensive Heart Failure Center (CHFC), University Hospital Würzburg, Würzburg, Germany; 9 Department of Internal Medicine I, University Hospital Würzburg, Würzburg, Germany; 10 Department of Cardiology, Erasmus MC, Rotterdam, The Netherlands

**Keywords:** Diabetes, Chronic kidney disease, Oxidative stress, Cardiac dysfunction, Mitochondria, Coronary microcirculation

## Abstract

Both diabetes mellitus (DM) and chronic kidney disease (CKD) predispose to cardiac remodeling and coronary microvascular dysfunction, which is proposed to be mediated through increased oxidative stress. To link oxidative stress and cardiac remodeling in DM and CKD, CKD was induced in genetically modified DM swine (*INS*^C94Y^ transgenic) at 10–12 weeks of age via renal microembolization, while non-embolized DM and wild-type (WT) swine served as controls. Compared to WT, 1) DM animals displayed modest LV dilation and a slight decline in ejection fraction, with increased end-systolic pressures and coronary blood flow. Addition of CKD did not further aggravate these alterations, but increased the pressure and diastolic wall stress compared to WT. 2) Proteomic analysis revealed enrichment in metabolic pathways involving fatty acids and glutamate, thus highlighting substantial metabolic reprogramming in both DM and DM_CKD groups. Re-analysis of proteomic data from human HFpEF patients showed differential regulation of similar pathways as well as anti-oxidant enzymes. 3) Isolated mitochondrial respiration was reduced in DM and DM_CKD hearts across multiple substrates (fatty acids, pyruvate-malate, glutamate-malate, and succinate), implicating broad mitochondrial dysfunction. 4) DM_CKD animals showed heightened oxidative stress in both coronary vasculature and myocardium as compared to both WT and DM [13 ± 3 (WT), 14 ± 3 (DM) and 39 ± 10% positive nuclei (DM_CKD)]. 5) LV-interstitial fibrosis was increased in DM_CKD (3.72 ± 0.50%) vs WT (1.70 ± 0.29%), with DM having an intermediate phenotype (2.82 ± 0.37%). Thus, even mild CKD in the presence of DM is accompanied by oxidative stress and ECM deposition. Our findings highlight the critical role of CKD in accelerating cardiac pathology and underscore the importance of targeting the cardiorenal axis in future therapeutic strategies.

**Supplementary Information:**

The online version contains supplementary material available at 10.1007/s00395-025-01153-6.

## Introduction

Heart failure (HF) is a systemic and syndromic disease in which various comorbidities contribute to the development and progression of the disease and vice versa, HF can predispose to the development of such comorbidities [9, 32, 40]. Among the most common comorbidities are metabolic diseases, such as obesity and diabetes mellitus (DM), but also chronic kidney disease (CKD) [57, 62]. The main factors that contribute to the systemic character of HF are neuroendocrine activation, inflammation, and alterations in metabolism [33]. In addition, hemodynamic alterations, such as increased cardiac pre- and afterload, contribute to mechano-energetic uncoupling in HF, especially with preserved ejection fraction (HFpEF), which results in mitochondrial oxidative stress as an important upstream mediator of cardiac remodeling and dysfunction [2].

In particular, the interplay of diabetes, CKD, and HF has received increasing attention in recent years [9], also spurred by emerging therapies that improve the outcome of all three of these entities. CKD is a global health burden that significantly increases cardiovascular morbidity and mortality, particularly in individuals with DM [18]. The combined effects of insulin resistance, metabolic dysregulation, and hyperglycemia further increase the risk of cardiovascular events in DM patients with CKD [37]. The synergistic effect of these conditions on the cardiovascular system has been termed cardiovascular-kidney-metabolic syndrome (CKM). Patients with CKM experience a spectrum of functional and structural cardiac abnormalities, eventually leading to diastolic dysfunction, myocardial fibrosis, and left ventricular (LV) hypertrophy [40]. One of the key mechanisms underlying this increased cardiovascular risk is oxidative stress, which plays a central role in the pathogenesis of HF in general, and in particular in cardiac dysfunction in CKD and DM [2, 22, 47, 54].

Oxidative stress arises from an imbalance between reactive oxygen species (ROS) production and elimination by endogenous antioxidant defense mechanisms [68]. Chronic hyperglycemia in DM disrupts normal mitochondrial function leading to excessive ROS generation, advanced glycation end-product (AGE) formation, and activation of pro-inflammatory pathways [29]. Both oxidative stress [7, 62] and mitochondrial dysfunction [20] contribute to endothelial dysfunction, myocardial remodeling, and impaired cardiac contractility, ultimately leading to diabetic cardiomyopathy and HF. In CKD, oxidative stress is further amplified due to increased inflammation, retention of uremic toxins, and dysregulated redox homeostasis. The interplay between oxidative stress and inflammation triggers myocardial interstitial collagen deposition [12]. In addition, the uremic milieu in CKD promotes endothelial dysfunction, impairs nitric oxide bioavailability, and induces capillary rarefaction and microvascular dysfunction, even prior to overt impairment of cardiac function, but eventually contributing to cardiac dysfunction and increased cardiovascular mortality [5, 7, 28, 63]. In our previous work, we observed that in a broad array of mouse [55, 69] and swine [54] models, the induction of CKD alone induces a pro-oxidative milieu, but without the development of cardiac dysfunction. Therefore, it is important to investigate *i)* the impact of CKD on the heart in larger animals and *ii)* in a more clinically relevant scenario, where other (metabolic) comorbidities, such as DM, co-exist (i.e., the concept of a “second hit”).

Here, we tested the hypothesis that CKD superimposed on DM exacerbates cardiac pathology primarily through the activation of cardiorenal signaling pathways that heighten oxidative stress and accelerate adverse structural remodeling**,** even prior to overt impairment of cardiac function. Furthermore, we aimed to explore early myocardial changes induced by detrimental cardiorenal signaling pathways. To this end, we employed a swine model with DM and CKD, and in a comprehensive approach, combined unbiased proteomic assessment of changes in myocardial signaling pathways with direct measurements of cardiac and coronary microvascular function in vivo, mitochondrial function ex vivo, and detailed histological and molecular assessment of myocardial fibrosis and oxidative stress. To underscore the translational value of our findings for patients with CKD, we validated our data by re-analyzing previously published cardiac proteomic data from patients with heart failure with preserved ejection fraction (HFpEF) that had both DM and CKD vs healthy donors [25].

## Methods

All animal experiments were approved by the Regierung von Oberbayern (ROB-55.2–2532.Vet_02-19–163) and were performed in accordance with the German Animal Welfare Act and Directive 2010/63/EU of the European Parliament on the protection of animals used for scientific purposes.

### Induction of diabetes and CKD

Twenty-six 12-week-old *INS*^C94Y^ transgenic (*INS*^C94Y^) pigs on a German Landrace background and fifteen age-matched wild-type (WT) littermates of either sex entered this study [51]. As previously described, *INS*^C94Y^ pigs gradually develop a diabetic phenotype after birth, because they carry a mutation in the insulin gene, causing insulin misfolding in the endoplasmic reticulum (ER), ER stress leading to beta-cell loss, defective insulin secretion, and persistent hyperglycemia. This model recapitulates key features of mutant *INS* gene-induced diabetes mellitus of the young (MIDY) in which the beta-cell function of the pancreas deteriorates early in life rather than typical type 2 diabetes mellitus (T2DM), which is generally characterized by insulin resistance and relative insulin deficiency [6, 51]. Blood glucose levels were measured on a weekly basis and insulin treatment (Lantus, Novorapid) was started when glucose levels rose above 300 mg/dL and adjusted to maintain glucose levels at this dysregulated level of approximately 300 mg/dL (Table S8). In eleven *INS*^C94Y^ swine, CKD was induced by microembolization of afferent glomerular arterioles in both kidneys, at 12 weeks of age (DM_CKD group) [54, 56]. Swine were sedated i.m. with ketamine 10% (20 mg/kg) (WDT, Garbsen, Germany), azaperone (10 mg/kg) (Stresnil, Elanco, Bad Homburg, Germany) and atropine sulfate (2 mg/kg) (Eifelfango, Neuenahr, Germany) against salivation, followed by midazolam (0.5 mg/kg i.v.) (Ratiopharm-Teva, Ulm, Germany). Swine were intubated and artificially ventilated (Primus, Dräger, Lübeck, Germany) with a mixture of O_2_ and air (1:3). Anesthesia and analgesia were maintained by 2–3% (vol/vol) sevoflurane (Sevorane, AbbVie GmbH, Ludwigshafen, Germany) and fentanyl (5–10 μg/kg/h i.v.) (Fentadon, Dechra, Aulendorf, Germany), respectively. A Swan-Ganz catheter (131F7, Edwards Lifesciences, Irvine, USA) was advanced in both renal arteries, the balloon was inflated and 75 mg of polyethylene microspheres (38–42 μm, Cospheric, Santa Barbara, CA, USA) were infused separately into each kidney. Thereafter, the carotid artery was ligated (only in the CKD induced animals) and the wound was closed.

### Hemodynamic assessment

At 8–9 months of age, animals were sedated as described above and anesthetized using propofol (7 mg/kg/h) and fentanyl (0.005–0.010 mg/kg/h). Echocardiography and hemodynamic measurements were performed as previously described [54]. In short, echocardiography was performed (Esaote, MyLabX8vet, Neufahrn, Germany) to assess wall thickness of the lateral wall (WTL) and septum (WTS) as well as left ventricular (LV) dimensions in M-mode, at systole (LVDes) and diastole (LVDed) as well as to measure the ratio of the rate of early (E) and late (A) diastolic LV filling as an index of diastolic function (E/A). Fractional shortening (FS) was calculated as (LVDed-LVDes)/LVDed, and wall-to-lumen ratio was calculated as (WTL + WTS)/LVD. For right and left heart catheterization, a 11F venous sheath (Terumo, RS*C11N10NR, Eschborn, Germany) and a 9F arterial sheath (Cordis, 504-609X, Miami Lakes, USA) were placed in the right external jugular vein and left internal carotid artery, respectively. The latter was connected to a pressure transducer to continuously monitor arterial blood pressure and heart rate.

Under fluoroscopic guidance (Ziehm Vision, Nuremberg, Germany), a Swan-Ganz catheter (131F7, Edwards Lifesciences, Irvine, USA) was introduced and progressed into the pulmonary artery via the venous sheath to measure the pressure in the pulmonary artery (PA) and the pulmonary capillary wedge pressure (PCWP). The cardiac output was assessed by thermodilution.

Using the arterial access sheath, a pressure–volume loop catheter (FDH-7018B-E245A, Transonic, Ithaca, USA) was placed in the LV, and PV loops were recorded using LabChart Pro (ADInstruments, Oxford, United Kingdom). Ventilation was briefly halted to obtain baseline PV loops as well as PV loops during preload reduction with a 14F Fogarty balloon (62080814F, Edwards Lifesciences, Irvine, USA) positioned in the inferior vena cava just below the diaphragm. Approximately 10 cardiac cycles recorded from the start of the occlusion were analyzed to obtain end-systolic and end-diastolic LV volumes (Ves, Ved) and pressures (Ped, Pes), and to calculate the end-diastolic pressure–volume relationship, end-systolic pressure–volume relationship, ejection fraction, as well as cardiac efficiency (ratio of stroke work and pressure–volume area). Because DM and DM-CKD animals were generally smaller than WT controls, all volumes and LV weight were indexed to body weight to allow comparison. Systolic and diastolic wall stress were calculated as Ves*Pes/LVweight and Ved*Ped/LVweight.

Subsequently, the thorax was opened, and a flow probe (3PSB, Transonic, Ithaca, USA) was placed around the proximal left anterior descending (LAD) coronary artery and connected to a computer using a perivascular flow module (TS420, Transonic, Ithaca, USA) and amplifier (16/35, ADInstruments, Oxford, United Kingdom). Baseline coronary blood flow (CBF-BL) was measured using LabChart Pro. An 6F angiocatheter (670–082-0E, Cordis, Miami Lakes, USA) was introduced via the arterial sheath into the LAD to infuse the vasodilator adenosine (50 μg/kg/min i.c., 01890, Merck, Taufkirchen, Germany) until maximum coronary blood flow (CBF-max) was achieved. Coronary flow reserve (CFR) was calculated as (CBF-max-CBF-BL)/CBF-BL. Heart samples were obtained from LV, snap-frozen in liquid nitrogen, and stored at -80 °C until further processing.

### Real-time quantitative PCR

30 mg of snap-frozen LV subendocardial tissue was homogenized and mRNA was extracted using the RNeasy Fibrous Tissue Mini kit (Qiagen, Hilden, Germany). cDNA was synthesized using 1000 ng of mRNA and a cDNA kit (M1661, ThermoFisher, Waltham, USA). Target genes were normalized against *HPRT* and *GAPDH* using the StepOne software (Applied Biosystem CA, USA). Relative gene expression was calculated using the delta–delta Ct method. The primers are listed in Table S7.

### Western blots

Subendocardial left ventricular tissue samples were homogenized in RIPA buffer supplemented with protease and phosphatase inhibitor cocktail. 30 µg of protein lysates were loaded in precast protein gels (4–20% gradient gel, Bio-Rad) and transferred to a PVDF membrane (Trans-Blot Turbo Mini 0.2 µm PVDF Transfer Pack, Bio-Rad). Membrane was blocked in 5% milk in PBST and then incubated with primary antibody overnight (eNOS/peNOS(Ser1177)/VEGF 1:1000; GAPDH – 1:10,000) and secondary antibody (1:5000) in 5% milk in PBST. The images were captured in iBright CL750, and quantification of bands by densitometry analysis was conducted in Image J Studio software. The antibodies are listed in Table S7.

### Enzyme-linked immunosorbent assays

To study the degree of kidney damage caused by renal embolization, neutrophil gelatinase-associated lipocalin (NGAL) and creatinine levels were determined in urine samples obtained after sacrifice using ELISA (ab207924, Abcam, Berlin, Germany and 502,330 Cayman Chemical, Michigan, USA, respectively), according to the manufacturer’s instructions. Insulin levels were determined in the swine plasma obtained at the time of sacrifice without any dilution according to manufacturer’s instructions (10–1221-01 Porcine Insulin ELISA kit, Mercodia, Uppsala, Sweden).

### Measurement of lipid peroxidation, glutathione peroxidase activity, total urinary protein and insulin

To assess lipid peroxidation (4-Hydroxynonenal (4-HNE)), 50 mg of LV sample (endocardium-anterior) was suspended in a lysing tube (845-CS-1140250, Innuscreen GmbH, Berlin, Germany) with 750 µL RIPA buffer (89,901, ThermoFisher, Waltham, USA) and homogenized using a homogenizer (Speedmill Plus, Analytik Jena GmbH, Jena, Germany). Subsequently, 4-HNE protein adducts were measured using a colorimetric assay (ab238538 Abcam, Berlin, Germany), according to the manufacturer’s instructions. Glutathione peroxidase (GPX) activity in serum was spectrophotometrically detected using a GPX assay kit (Abcam, ab102530). The kit is based on reducing the oxidized glutathione coupled with NADPH oxidation. The reduction in NADPH was determined at 340 nm. The absorbance was read at 340 nm after 20 min. Total protein in urine was determined by the Bradford assay (23,246 ThermoFisher, Waltham, USA) according to manufacturer’s instructions and normalized to urinary creatinine to correct for DM-induced polyuria.

### Mitochondrial measurements

Isolation of mitochondria from freshly excised left ventricular tissue was performed as previously described [34, 67]. After excision, several 200-mg portions of the left ventricle were washed in ice-cold isotonic isolation solution (IS; in mM: sucrose 75, mannitol 225, HEPES 2, EGTA 1, pH 7.4) and separated from non-myocardial tissue. Then the portions were transferred to a 5-mL homogenizer (Teflon pestle) and manually homogenized in two steps for 7 min each in 1 mL isolation solution, containing additionally BSA (4 mg/mL) and 0.16 mg/mL of proteinase. The homogenate was centrifuged at low speed (480 g, 5 min, 4 °C), and the supernatant was further centrifuged (7700 g, 10 min) to obtain the mitochondrial pellet and the cytosolic fraction, and washed carefully twice in 1.4 mL mitochondrial suspension solution (MSS; like IS, but only 20 µM EGTA) and centrifuged (7700 g, 10 min). The pellets were finally resuspended in 100–200 µL of MSS as appropriate. Protein concentration of the final mitochondrial suspension was measured using the Lowry method, and OD750 equivalents corresponding to 400 μg of isolated mitochondria were used in high-resolution respirometry, as described previously [39]. Oxygen consumption was assayed at 37 °C with an Oxygraph-2 k high-resolution respirometer, and DatLab 7.4 was used for data acquisition and analysis (Oroboros Instruments, Innsbruck, Austria). Three mitochondrial respiration protocols were used to quantify oxygen consumption rate upon supplementation of pyruvate (for carbohydrate metabolism) or fatty acids as a fuel. In the first protocol, measurements of complex I and II activity were performed using 5 mM Na-pyruvate and 5 mM Na-malate. The metabolites were added as reduced substrates after initially recording residual oxygen consumption resulting in leak respiration, followed by increasing concentrations of ADP (0.03, 0.1, 0.3, 1 mM). Then 5 mM K-glutamate was added. Complex I was inhibited by 0.5 µM rotenone to prevent reverse electron flux, followed by addition of 10 mM succinate, a complex II substrate. Finally, oxygen consumption coupled to ADP phosphorylation was inhibited by adding 1.25 µM oligomycin, followed by titration with 10 µM DNP to determine uncoupled respiration State 3 u. The second carbohydrate protocol included the addition of 5 mM glutamate and malate followed by the addition of 1 mM ADP, 1.25 µM oligomycin, and DNP steps. In the ‘fatty acid’ protocol, respiration was measured using 1 mM carnitine, 3 mM malate, 10 µM palmitoyl-CoA, 10 µM oleoyl-L-carnitine. Similar to the ‘carbohydrate’ protocol, increasing amounts of ADP, 1.25 µM oligomycin, and 10 µM DNP were added. Mitochondrial membrane potential was simultaneously probed using 1 µM TMRM and Smart Fluo-Sensor Green, as described before [31, 39].

To quantify mitochondrial hydrogen peroxide (H_2_O_2_) emission, two distinct protocols were carried out. Following the addition of mitochondria, 5 U/mL SOD, 1 U/mL HRP, and 10 µM Amplex Red were added, followed by 1 mM carnitine, 3 mM malate, 10 µM palmitoyl-CoA, 10 µM oleoyl-carnitine, or 5 mM pyruvate and malate, 1 mM ADP and antimycin A (1.5 µM) were added. The data were acquired and analyzed using DatLab V7.4 software.

### Proteomics

Sample preparation (WT N = 5, DM N = 5, DM_CKD N = 4) and proteomics were performed as in our previous study [54]. Frozen left ventricular heart tissue samples were cryopulverized. The samples were digested sequentially, first using Lys-C alone and then adding additionally porcine trypsin. For LC–MS/MS analysis, an UltiMate 3000 nano-LC system connected online to a Q Exactive HF-X instrument (Thermo Fisher Scientific, Waltham, USA) was used. 1 μg of the digest was injected and MS spectra were acquired with data independent acquisition. Raw data were processed using DIAN-NN (1.8.1) and the NCBI RefSeq Sus scrofa database (v.7–5-2020). All statistical analyses and data visualization were performed using an R package which can be found at https://github.com/bshashikadze/pepquantify. Proteins were quantified using the MS-EmpiRe algorithm [4]. Revigo was used to get rid of redundant terms in the pre-ranked STRING analysis [58]. The raw mass spectrometry data and DIA-NN output have been deposited to the ProteomeXchange Consortium via the PRIDE [46] partner repository with the dataset identifier PXD065331.

To enhance translational relevance, we reanalyzed publicly available human myocardial proteomic data from a recent study by Jani et al. [25]. Normalized proteomic datasets were downloaded and subjected to differential expression analysis using the limma package in R.

In the original publication, principal component analysis (PCA) identified two distinct clusters among patients with heart failure with preserved ejection fraction (HFpEF). For the present analysis, we focused on the cluster comprising patients with both diabetes mellitus (DM) and chronic kidney disease (CKD), defined by a glomerular filtration rate (GFR) < 75 mL/min/1.73 m^2^, N = 3. Among the donor control samples, two clusters were identified. Differential expression analysis comparing the DM + CKD cluster with Con cluster1 (no DM and GFR =  > 75 mL/min/1.73 m^2^, N = 4) revealed several significantly regulated proteins (Table S9). The second cluster (Con cluster2) had no GFR data for one patient and only two patients who met our criteria (no DM and GFR =  > 75 mL/min/1.73 m^2^). Due to low power in this group, we excluded it from our analysis. Functional enrichment and pathway analyses were performed using the STRING database to identify biologically relevant protein interaction networks. In addition, dot blot assays were employed to validate key oxidative stress–related proteins identified as differentially regulated (Table S9).

### Histology and immunohistochemistry

LV myocardial tissue and kidney samples were cut, fixated in Rotihistofix (Roth, P087.1), and transferred into 70% alcohol after 48 h. After that, the tissue was embedded in paraffin. 3-µm deparaffinized sections were stained with Picrosirius red staining solution (Polysciences, Picrosirius Red Stain Kit#24,901). The Picrosirius red (PR) staining was analyzed under polarized light, and the amount of birefringence was quantified in ten randomly chosen non-overlapping fields (× 200 magnification) using QuPath software. For immunostaining, 3 µm deparaffinized LV sections were boiled in citrate buffer, pH 6.0, for antigen retrieval. The sections were incubated with primary antibodies for 8-hydroxy-2'-deoxyguanosine (1:1000 dilution; Abcam, #ab48508) CD31 (1:100 dilution; Thermo Scientific, MA5-32,321) and WGA (1:1000 dilution; Thermo Scientific, W21404) overnight. The following day, the sections were incubated in species-specific secondary antibodies (1:500 dilution; Abcam, goat anti-mouse ab150113 or goat anti-rabbit ab150080) for 2 h, and sections were mounted with DAPI for nuclear staining. The stained sections were quantified in ten randomly chosen non-overlapping fields (× 400 magnification) using ImageJ software.

### Statistical analysis

Statistical analysis was performed using a one-way or two-way Analysis of Variance for Repeated Measurements (post hoc Tukey test for multiple comparisons) in Graphpad Prism (v10). All data passed the normality test using the Shapiro–Wilk test, and data are shown as mean ± SEM. Because there were no differences between male and female swine, data from both sexes were pooled.

## Results

**Fig. 1:**
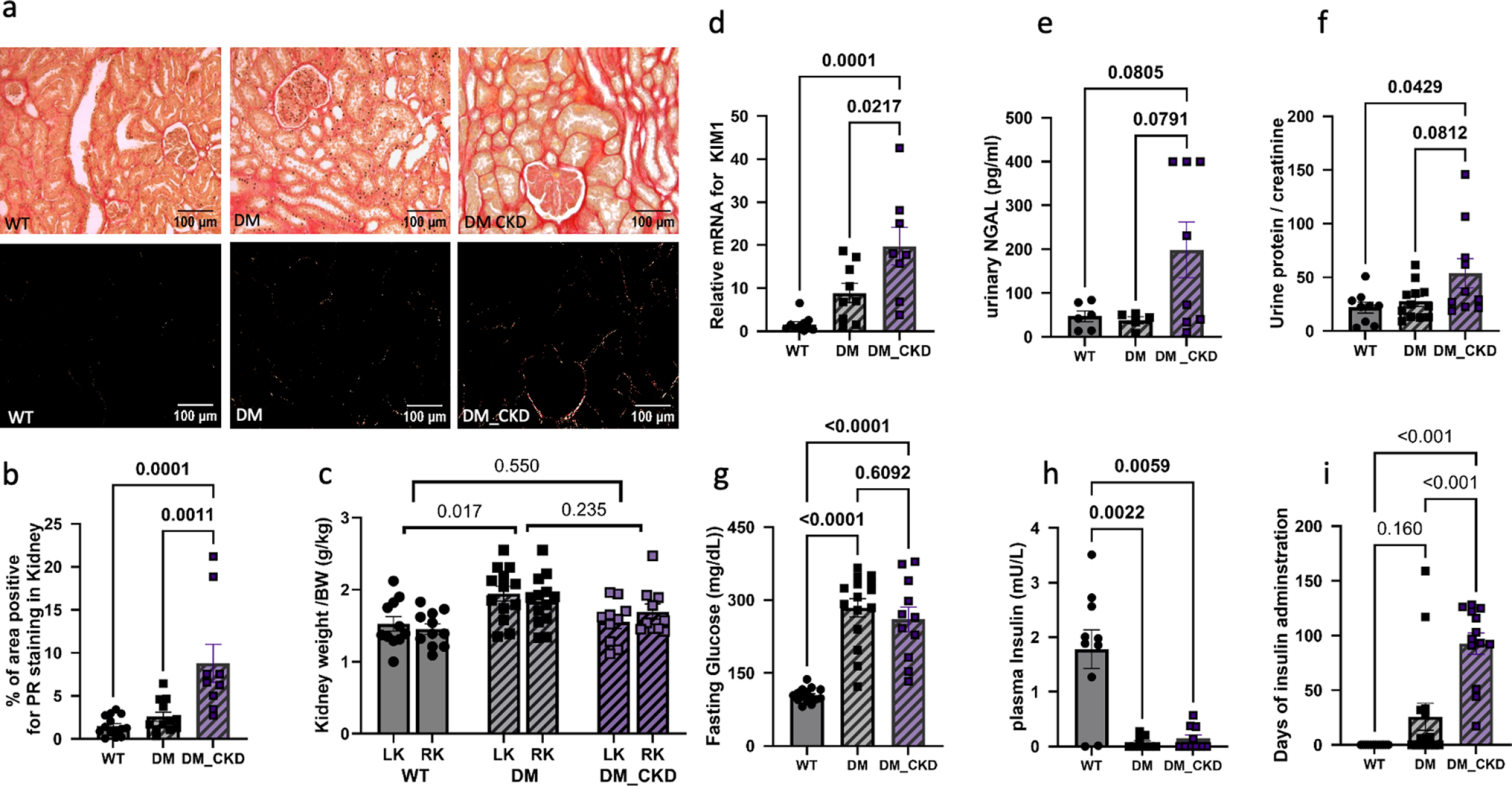
Induction of CKD post renal artery embolization. **a**, **b** Representative images of Picrosirius red (PR) staining in kidneys and quantification of the percentage of area positive for PR staining under polarizing light microscopy in kidneys from WT, DM, and DM_CKD swine. N = 14 in WT, N = 12 in DM, and N = 9 in DM_CKD. **c** Kidney weight/total body weight (BW) in left and right kidney. N = 15 in WT and DM, N = 11 in DM_CKD. **d**, **e** Relative mRNA quantification of kidney injury molecule 1 (*KIM1*) and urinary neutrophil gelatinase-associated lipocalin (NGAL) measured via ELISA from kidneys of WT, DM, and DM_CKD swine. N = 10 in WT, N = 9 in DM, and N = 8 in DM_CKD. **f** Urinary protein to creatinine ratio from WT, DM, and DM_CKD swine. N = 10 in WT, N = 12 in DM, and N = 10 in DM_CKD. **g**–**i** Blood fasting glucose, plasma fasting insulin level and days of insulin administration in N = 15 in WT, N = 15 in DM, and N = 10 in DM_CKD. Values are mean ± SEM. *p* values were calculated by ordinary one-way ANOVA with Tukey correction

Three groups of swine were studied: wild-type controls (WT), diabetic (DM), and DM with superimposed CKD (DM_CKD). DM alone did neither result in significant changes in renal fibrosis (Fig. 1a,b), nor in changes in markers of kidney injury (Kidney injury molecule 1, KIM1; Fig. 1d), NGAL (a tubular injury marker, Fig. 1e) or urinary protein to creatinine ratio (UPCR, Fig. 1f), although kidney weight was significantly increased (Fig. 1c). In contrast, CKD induced via renal embolization in addition to DM increased renal fibrosis, KIM1, NGAL, and UPCR in comparison to WT and DM alone (Fig. 1a–f), although the increase in NGAL and UPCR in DM-CKD vs DM alone failed to reach statistical significance (both *p *= 0.08). DM resulted in a significant reduction in fasting insulin levels (Fig. 1h) that were accompanied by an increase in fasting glucose levels (Fig. 1g). Intriguingly, DM_CKD animals required significantly more insulin to titrate their plasma glucose levels to 300 mg/dL (Fig. 1i), suggesting that the kidney injury aggravated insulin resistance and DM (Table S8).

### Hemodynamic alterations post CKD

**Fig. 2:**
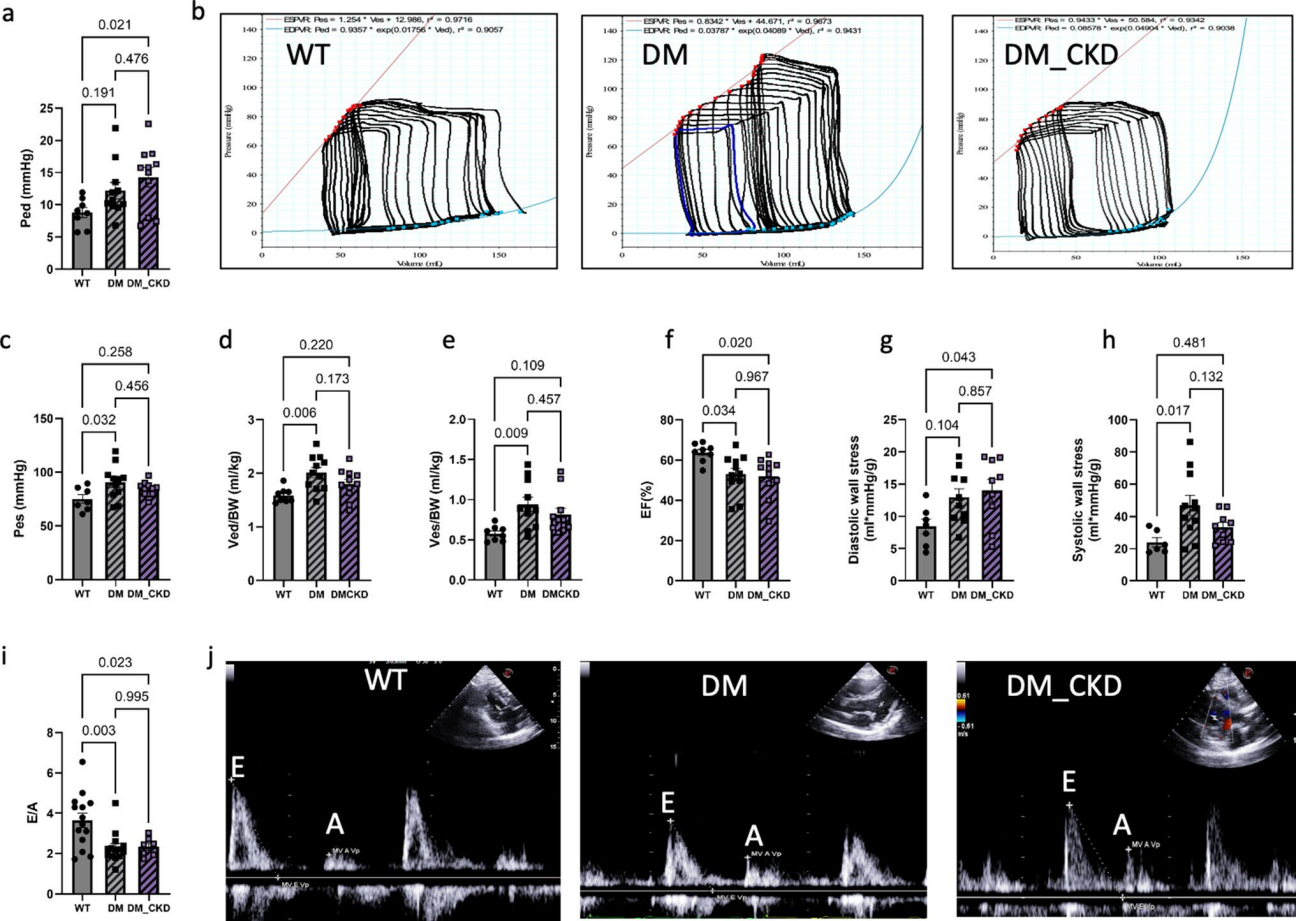
Cardiac function measured by pressure–volume (PV) loop (**a**–**h**) and ultrasound (**i**–**j**). Pressure–volume loop parameters—**a** left ventricular end-diastolic pressure (Ped); **b** representative images of pressure–volume loops in WT, DM, and DM_CKD swine; **c** left ventricular end-systolic pressure (Pes); **d**, **e** left ventricular end-diastolic volume (Ved) and end-systolic volume (Ves) normalized to body weight (BW); **f** ejection fraction (EF); **g** end-diastolic wall stress (calculated as Ped * Ved / LV mass); **h** end-systolic wall stress (calculated as Pes * Ves / LV mass); N = 8 (WT), N = 11 (DM), and N = 11 (DM_CKD). Echocardiography to assess **i** the ratio of early (E) to late (A) diastolic filling rates at the mitral valve and **j** representative images of the E/A ratio in N = 14 (WT), N = 14 (DM), and N = 7 (DM_CKD).Values are mean ± SEM. *p* values were calculated by ordinary one-way ANOVA with Tukey’s correction

Heart rate (HR), cardiac output (CO), and stroke volume (SV) were similar in all groups (Table S1). However, pressure–volume (PV) loop and ultrasound measurements revealed alterations in cardiac function that were directionally similar for DM and DM_CKD, although specific alterations sometimes failed to reach significance in either group. Typical examples of PV-loop and ultrasound images are shown in Fig. 2. A decrease in E/A (Fig. 2i) suggestive of diastolic dysfunction was accompanied by an increase in LV end-diastolic pressure (Ped) in DM_CKD (Fig. 2a), while LV end-diastolic volume (Ved) was significantly increased in DM (Fig. 2d). This led to a decrease in the wall-to-lumen ratio (Fig. S1) and an increase in diastolic wall stress, which was statistically significant in DM_CKD as compared to WT only (Fig. 2g).

Although Pes, Ves, and systolic wall stress were significantly increased only in DM compared to WT (Fig. 2c,e,h), wall-to-lumen ratio (Fig. S1), ejection fraction (EF; Fig. 2f) and fractional shortening (Fig. S1) were slightly decreased in both DM and DM_CKD as compared to controls, suggesting mild systolic dysfunction, but nominally still in the range that can be considered HFpEF (LVEF ≥ 50%). The overall rightward shift in the PV loops resulted in reduced cardiac efficiency (from 0.71 ± 0.08 in WT to 0.52 ± 0.11 in DM; *p* = 0.003 vs WT; and 0.57 ± 0.12 in DM_CKD, *p* = 0.031 vs WT, *p* = 0.5 vs DM), indicating an increase in total myocardial work for the same stroke work. Altogether, these data indicate LV systolic and diastolic dysfunction in DM, consistent with the presence of diabetic cardiomyopathy with no or minor additional impact of CKD.

### Coronary flow and endothelial function

**Fig. 3:**
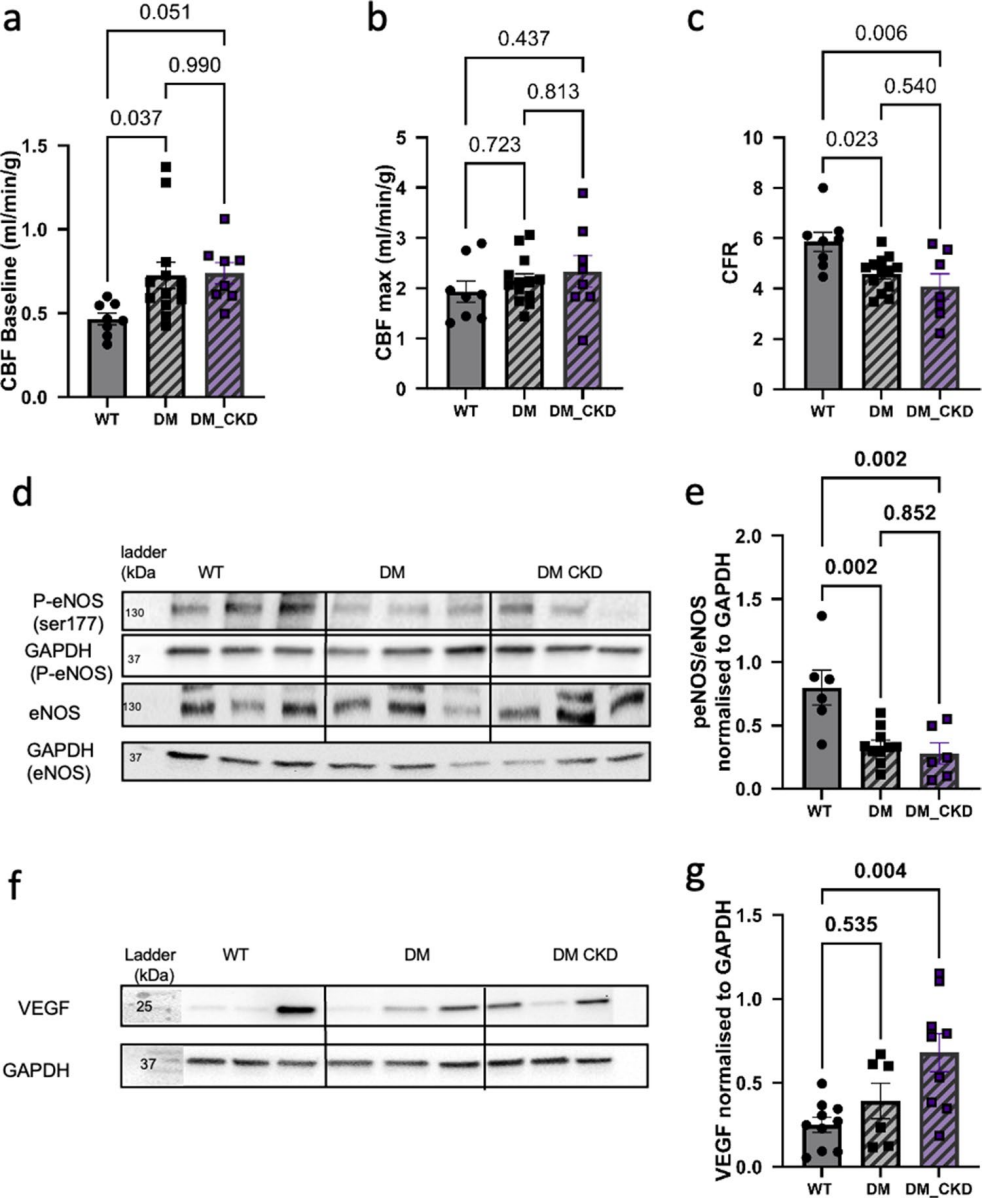
Coronary flow and endothelial function in left ventricular (LV) tissue in DM and DM_CKD animals. **a** Coronary blood flow at baseline; **b** coronary blood flow during maximal vasodilation induced by adenosine; **c** ratio of maximal flow to baseline flow (coronary flow reserve) in WT, DM, and DM_CKD groups. N = 7 in WT, N = 13 in DM, and N = 8 in DM_CKD; **d**, **e** representative blots and quantification of eNOS and phospho-eNOS (ser177), respectively, in LV endocardial tissue from WT, DM, and DM_CKD groups (N = 6 WT, N = 9 DM, N = 6 DM_CKD); **f**, **g** representative blot and quantification of VEGF protein in LV endocardial tissue from WT, DM, and DM_CKD groups (N = 10 WT, N = 6 DM, N = 9 DM_CKD).Values are mean ± SEM. *p* values were calculated by ordinary one-way ANOVA

In accordance with our previous work [54, 63, 65] as well as with the decrease in cardiac efficiency, both DM and DM_CKD groups exhibited significantly increased baseline coronary blood flow (CBF) compared to WT controls, while adenosine-induced maximal CBF (CBF-max) remained unchanged across all groups (Fig. 3a,b). Hence, coronary flow reserve was significantly reduced in both DM and DM_CKD group relative to WT (Fig. 3c). Although this decrease in coronary flow reserve was driven mostly by the increased baseline CBF rather than a decrease in CMF-max, the ratio of p-eNOS (Ser1177) to total eNOS was decreased in both DM and DM_CKD as compared to WT, suggesting compromised endothelial nitric oxide signaling (Fig. 3d, e). The protein expression of vascular endothelial growth factor (VEGF) was significantly increased only in the DM_CKD group (Fig. 3f, g). These findings collectively suggest that diabetes plays the major role in dictating coronary flow, while CKD has an additive impact on angiogenic signaling.

### Proteomic changes in the heart following CKD in diabetes

**Fig. 4:**
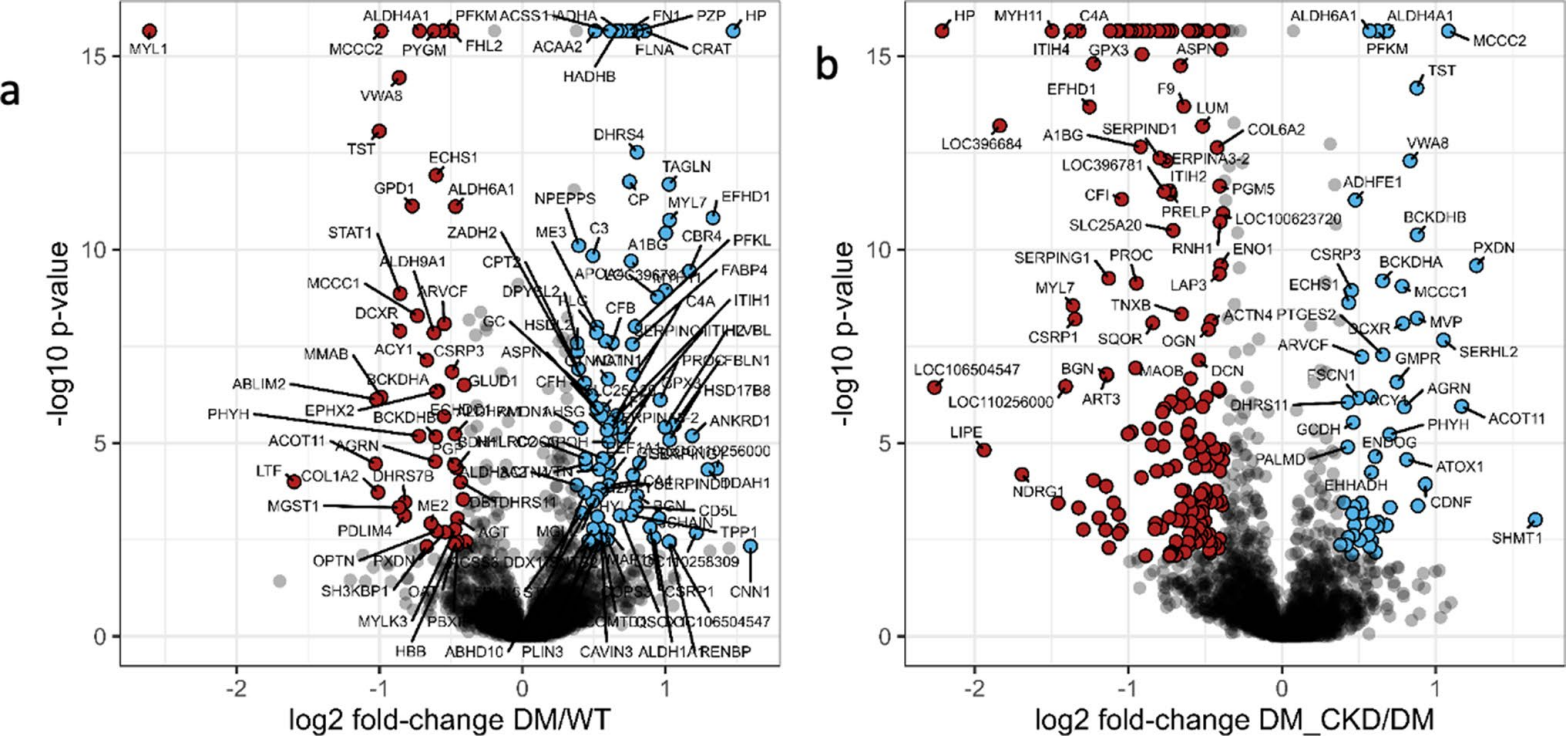
Proteomic analysis of LV myocardial tissue from WT, DM, and DM_CKD pigs. **a** Quantitative proteome changes are represented via volcano plots in DM vs WT and DM_CKD vs DM animals, respectively. Color-filled circles (blue—upregulated, red—downregulated) indicate differentially abundant proteins (Benjamini–Hochberg corrected *p* value < 0.05 and fold change > 1.3). N = 5 for WT and DM, and N = 4 for DM_CKD pigs. All gene name descriptions can be found in the Human Gene Nomenclature committee website https://www.genenames.org/

**Fig. 5:**
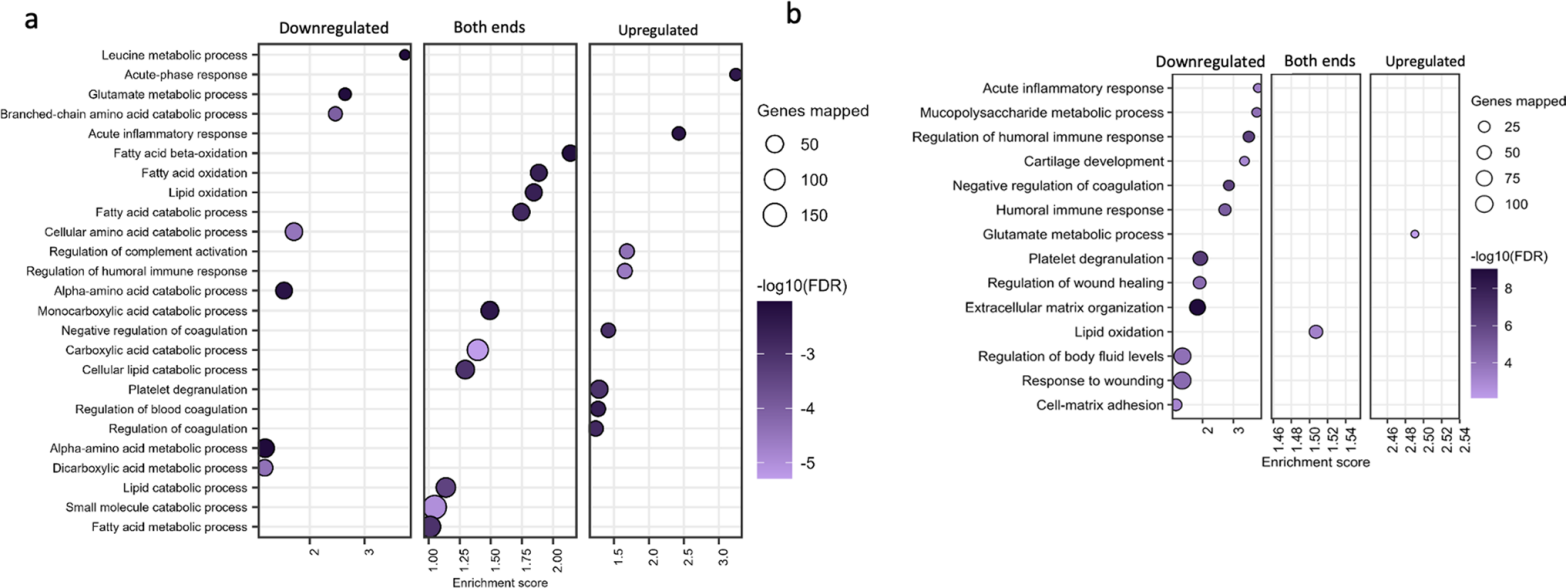
Functional pathway analysis of proteomic data from LV myocardial tissue in DM and DM_CKD. **a** Over-representation analysis using pre-ranked gene set enrichment analysis using STRING was employed to reveal biological processes related to differentially abundant proteins in DM vs WT and DM_CKD vs DM, respectively. Signed (based on fold change) and log-transformed *p* values were used as ranking metrics, and the false discovery rate was set to 1%. Size of the bubble indicates the corresponding number of differentially abundant proteins in the pathway (referred to as genes mapped in the figure), and color represents the significance of enrichment. N = 5 for WT and DM, and N = 4 for DM_CKD pigs

To investigate the molecular pathways differentially regulated in the myocardium of swine with DM and DM_CKD, we performed mass spectrometry-based proteomics on LV samples and detected 3500 proteins (Table S2). Differential abundance analysis revealed 136 proteins significantly altered between WT and DM, and 217 proteins between DM and DM_CKD groups (Benjamini–Hochberg corrected *p *value < 0.05, fold change ≥ 1.3), as visualized in volcano plots (Fig. 4a, b; Tables S3, S5). In the DM group compared to controls, we observed upregulation of proteins involved in the coagulation pathway (C3, C4a, CFB, F2) and contractile proteins (ACTN1, ACTN4, MYH11) alongside the alterations of mitochondrial proteins (CPT2, SLC25A, BDH1, ALDH6 among others). STRING-based pre-ranked functional enrichment analysis using the Gene Ontology (GO) biological processes database identified significantly enriched terms for DM vs. WT and DM_CKD vs. DM comparisons (enrichment factor > 1; Fig. 5a, b, Tables S4, S6). Consistent with the diabetic phenotype, changes in metabolic processes were observed, including downregulation of branched-chain amino acid metabolism and glutamate metabolism, while some proteins in pathways related to fatty acid metabolism were upregulated and others downregulated. Furthermore, upregulation of the acute-phase response, complement activation, and the humoral immune response denote a pro-inflammatory response.

In the comparison between DM_CKD and DM, mitochondrial proteins showed further alterations (MTCO2, SLC25A, ALDH9A), accompanied by decreased abundance of blood coagulation factors (F9, C9, C7), extracellular matrix remodeling proteins (COL6, FBN1, TGFB, PLG) as well as proteins involved in redox regulation (GPX3, PRDX1). Pathway analysis revealed that proteins related to acute phase response showed a downward trend in DM_CKD, suggesting that immune response was downregulated in DM_CKD compared to DM. Notably, proteins related to glutamate metabolism, which is essential for cardiac energy balance and oxidative stress adaptation, were downregulated in DM versus WT, but upregulated in DM_CKD compared to DM. The metabolic pathways related to lipid oxidation were also altered in DM_CKD vs DM (Fig. 5a, b, Table S4, S6).

### Proteome changes in human myocardium with diabetes and CKD

**Fig. 6:**
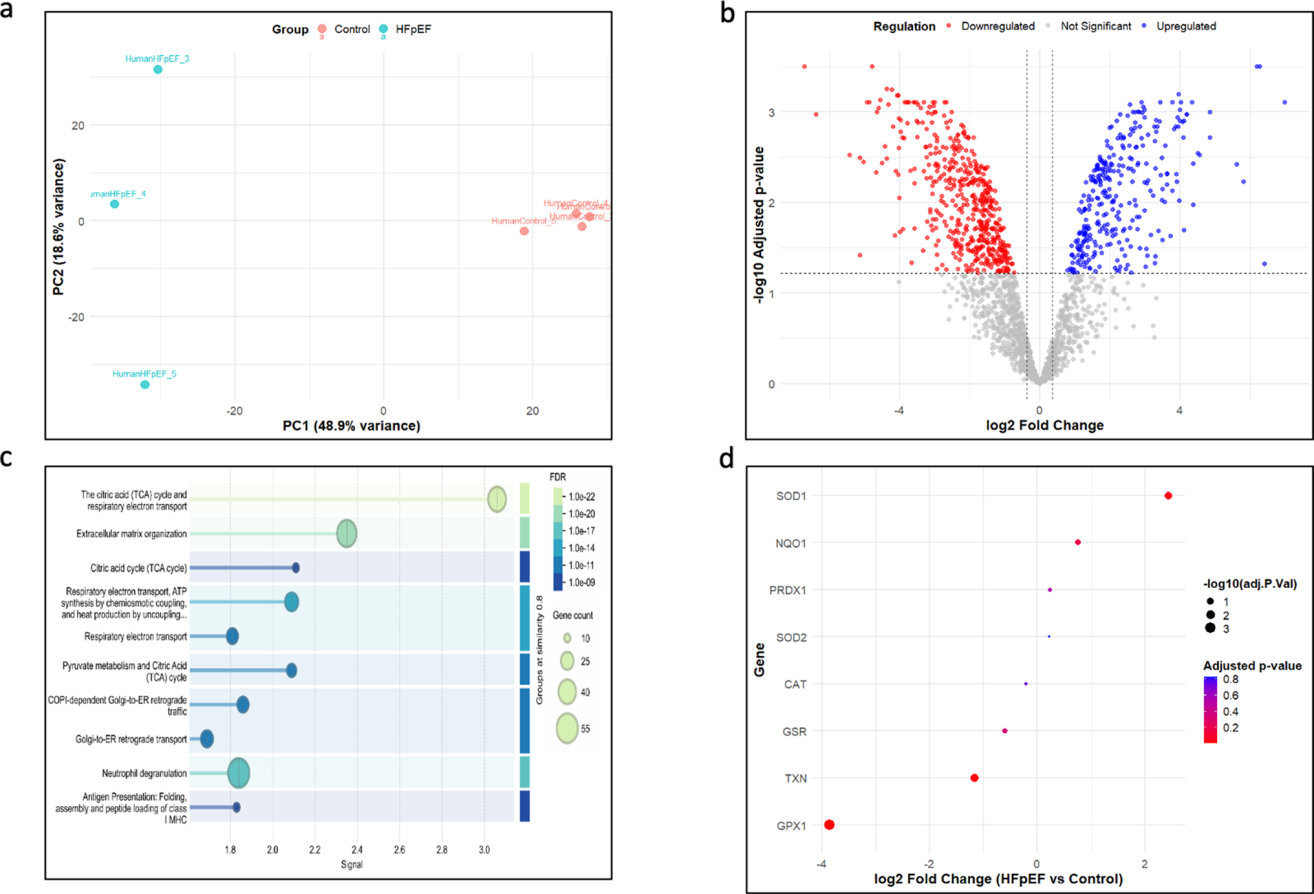
Proteomic analysis in human HFpEF patients with DM and CKD versus donor control. **a** PCA analysis of the groups HFpEF patients with DM and CKD versus donor control, **b** quantitative proteome changes are represented via volcano plots in HFpEF patients with DM and CKD vs donor controls, respectively. Color-filled circles (blue—upregulated, red—downregulated), **c** STRING pathway analysis on the differentially downregulated proteins between the HFpEF patients with DM and CKD vs donor controls, **d** dot plot analysis for specific genes involved in the oxidative stress pathway. Color of the bubble indicates the corresponding adj *p* value of the protein. N = 3 in HFpEF patients with DM and CKD and N = 4 in donor control. Benjamini–Hochberg corrected *p* value < 0.05 and fold-change > 1.3

Since DM and CKD are among the most common comorbidities in patients with heart failure, especially with HFpEF [57], we re-analyzed human myocardial proteomic data previously published by Jani et al. [25]. Principal component analysis showed distinct clustering of most of the patients with HFpEF and CKD, with differential regulation of 765 proteins represented with a volcano plot (Fig. 6 a, b). Notably, downregulated proteins were enriched in pathways related to the tricarboxylic acid (TCA) cycle and extracellular matrix (ECM) remodeling (Fig. 6c), closely mirroring the metabolic and structural changes observed in our DM_CKD swine model. Furthermore, a focused analysis of oxidative stress-related proteins via dot-plot indicated significant downregulation of glutathione peroxidase 1 (GPX1) in HFpEF and CKD hearts, whereas superoxide dismutase 1 (SOD1) was substantially upregulated (Fig. 6d).

### Mitochondrial dysfunction post CKD

**Fig. 7:**
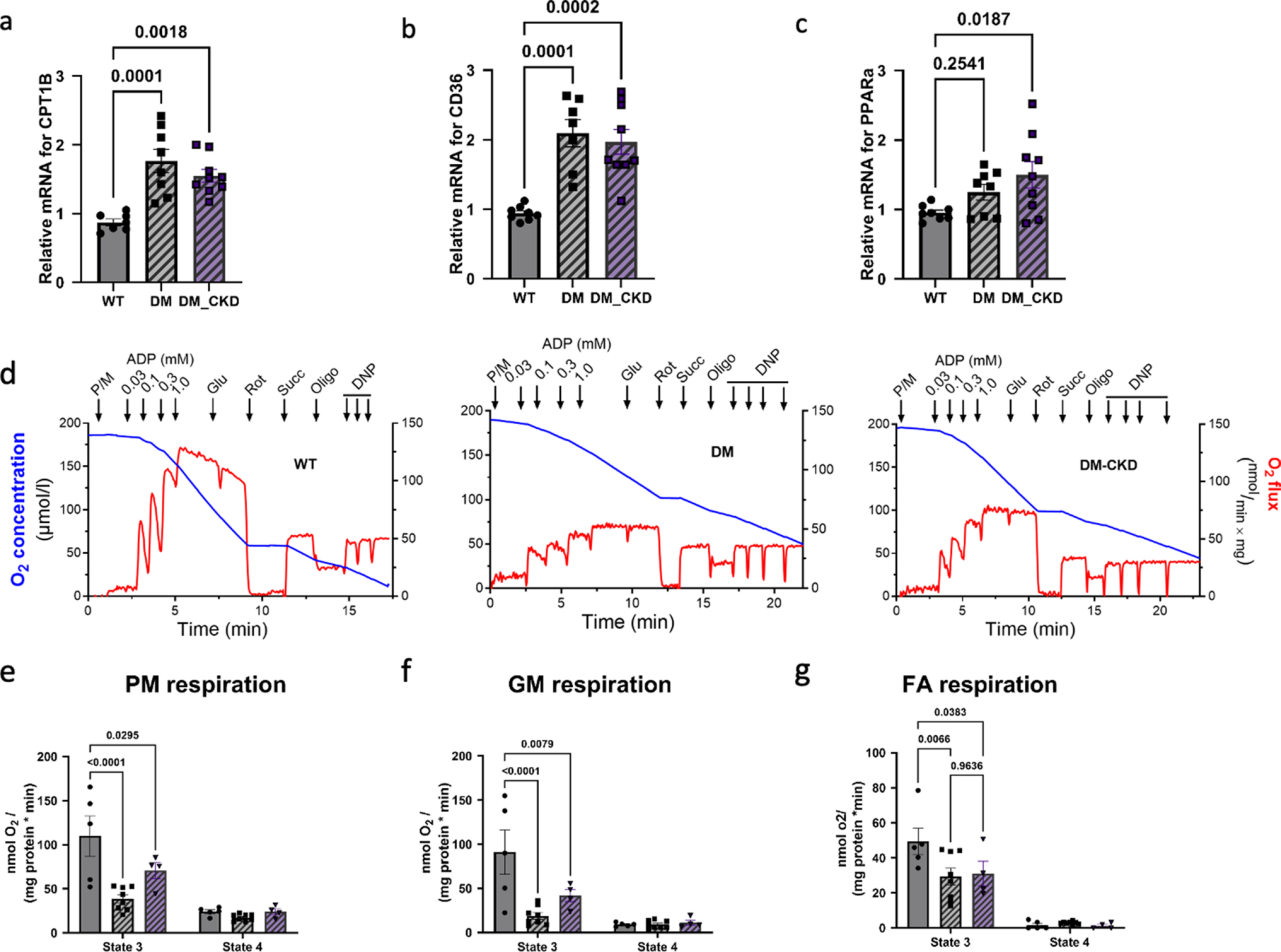
Fatty acid oxidation and mitochondrial respiration. **a**–**c** Quantitative RT-PCR of *CPT1B*, *CD36*, and *PPARa*, in the LV endocardial tissue in WT, DM, and DM_CKD groups. N = 8 for WT and DM group, and N = 9 for DM_CKD; **d** representative traces of oxygen conc. (blue) and oxygen flux (red) of isolated cardiac mitochondria from WT, DM, and DM_CKD animals supplied with pyruvate/malate and succinate as substrates. **e**–**g** O_2_ consumption of isolated cardiac mitochondria from WT, DM, and DM_CKD groups supplied with pyruvate and malate, glutamate and malate as well as fatty acids in presence of saturating ADP (State 3). The complex V inhibitor oligomycin was added to inhibit phosphorylation-coupled respiration (State 4). N = 5 for WT, N = 8 for DM, and N = 4 for DM_CKD pigs. *p* value by two-way ANOVA

To further explore the alterations in fatty acid oxidation and confirm the proteomic findings observed in LV tissues, we performed RT-PCR analysis on genes important for fatty acid uptake, transport, and oxidation—Peroxisome Proliferator-Activated Receptor alpha (*PPARa*), *CD36* and carnitine palmitoyltransferase I (*CPT1B*) [13]. *PPARa* was significantly increased only in the DM_CKD group, and the other two genes (*CD36* and *CPT1B*) showed significantly increased expression in both DM and DM_CKD groups, indicating enhanced fatty acid oxidation compared to the WT (Fig. 7a–c). Given the alterations in mitochondrial proteins in our proteomic data and the fact that mitochondria are the major site of fatty acid oxidation in cardiomyocytes, we assessed mitochondrial function via high-resolution respirometry in isolated mitochondria. Exemplary traces of a protocol using pyruvate and malate as metabolic substrates in the three treatment groups are shown in Fig. 7d. State 3 respiration, shown as the maximal rate of respiration in response to 1 mM ADP, in the presence of either pyruvate-malate, fatty acids, or glutamate-malate was significantly reduced in both DM and DM_CKD groups compared to WT, while State 4 respiration and membrane potential measured by TMRM were unchanged, indicating no relevant mitochondrial uncoupling (Fig. 7 e–g, Fig. S2 a–c). Also respiration with succinate (a complex II substrate) was depressed in both DM and DM_CKD compared to WT (Fig. S3a). In contrast, H₂O₂ emission during state 3 respiration (which most closely resembles the in vivo situation) was unchanged in DM and DM_CKD vs. WT mitochondria, while H_2_O_2_ emission in response to antimycin A, which blocks complex III of the respiratory chain and leads to an artificial maximal superoxide formation, was slightly decreased in DM and DM_CKD cardiac mitochondria when respiring on fatty acids or pyruvate/malate, respectively (Fig. S4b and c). Hence, decreased mitochondrial respiration with neither uncoupling nor increased ROS emission was induced by DM, but not further aggravated by CKD in addition to DM.

### Increased oxidative stress post CKD

**Fig. 8:**
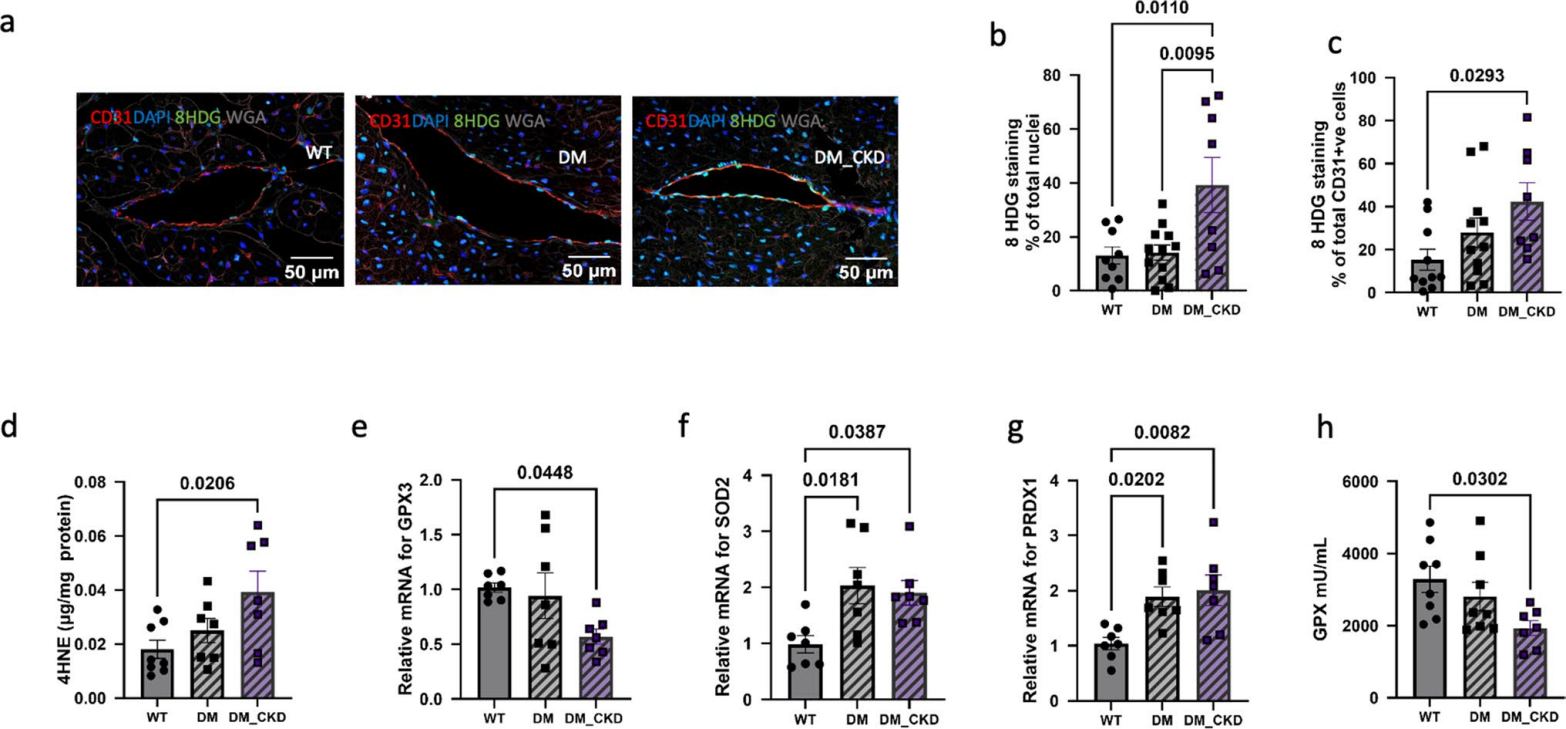
Oxidative stress in left ventricular (LV) tissue post-CKD. **a** Representative immunofluorescence images showing 8-hydroxy-2'-deoxyguanosine (8-HDG) staining in CD31-positive vessels in LV paraffin-embedded tissue from WT, DM, and DM_CKD groups. Original magnification, × 400. **b**, **c** Quantification of 8-HDG-positive nuclei in total LV tissue (**b**) and specifically in CD31-positive cells (**c**). Sample sizes: N = 9 (WT), N = 12 (DM), N = 8 (DM_CKD); **d** quantification of 4-hydroxynonenal (4-HNE) levels measured by ELISA to assess levels of lipid peroxidation in LV tissue homogenates; **e** quantitative RT-PCR analysis of antioxidant genes *GPX3*, *SOD2*, and *PRDX1* in LV endocardial tissue from WT, DM, and DM_CKD groups; **f** serum GPX levels measured by ELISA. Sample sizes: N = 7 (all groups for RT-PCR), N = 8 (WT), N = 7 (DM and DM_CKD) for ELISA. All values are presented as mean ± SEM. Statistical significance was assessed by ordinary one-way ANOVA

Since elevated fatty acid oxidation is known to increase oxidative stress in diabetic cardiomyopathy—a finding also supported by our proteomic data—we assessed several markers of oxidative stress. Immunostaining for 8-hydroxy-deoxyguanosine (8-HDG), a well-established marker of oxidative DNA damage, was significantly elevated in paraffin-embedded LV sections of DM_CKD, both in the myocardium and CD31-labeled coronary microvessels, while there was no change in DM (Fig. 8 a–c). In addition, we detected increased formation of 4-HNE adducts, a stable byproduct of lipid peroxidation and indicator of oxidative membrane damage (Fig. 8d), further substantiating enhanced oxidative stress in DM_CKD, but not DM hearts.

To further assess changes in anti-oxidant pathways, the expression of key enzymes was determined by real-time PCR. In line with our proteomic results, *GPX3* was significantly downregulated in DM_CKD, but not DM alone (Fig. 8e). We also detected a decrease in GPX activity in the serum of DM_CKD swine (Fig. 8h). Conversely, we observed an increased expression of *SOD2* and *PRDX1* (Fig. 8 f,g). Importantly, oxidative stress-related proteins in the human proteomic data of patients with HFpEF, DM, and CKD displayed similar patterns: GPX1 was downregulated, while SOD1 was upregulated, reflecting the redox imbalance identified in our animal study (Fig. 6d). Together, these complementary findings provide robust evidence of redox imbalance and oxidative injury contributing to myocardial pathology in the combined diabetic and CKD setting.

### ECM changes post CKD

**Fig. 9:**
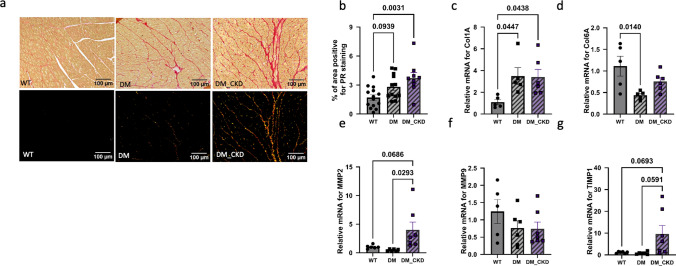
Remodeling in LV tissue post-CKD. **a** Representative images of Picrosirius red (PR) of the heart under bright field and polarizing light, respectively and **b** quantification of the area positive for PR staining under polarizing light; original magnification, × 200. N = 14 in WT, N = 12 in DM, and N = 9 in DM_CKD (**c**–**g**) Quantitative RT-PCR of *COL1A*, *COL6A*, *MMP2*, *MMP9*, and *TIMP1* mRNA in the LV endocardial tissue in WT, DM, and DM_CKD groups. N = 5 animals in WT and DM, N = 6 in DM_CKD. Values are mean + / − SEM. *p* value by ordinary one-way ANOVA

Finally, to ascertain the impact of increased oxidative stress and mitochondrial dysfunction on cardiac remodeling in the DM and DM_CKD groups, we assessed fibrosis in the LV tissue. The DM group showed a trend towards increased fibrosis, whereas the DM_CKD group exhibited significantly greater fibrosis than WT controls, as shown by PR staining (Fig. 9). Both diabetic groups had elevated *COL1A2* expression, while *COL6A1* expression was notably downregulated in the DM group. In addition, the mRNA for the ECM remodeling enzyme MMP2 was significantly upregulated in the DM_CKD group compared to DM alone and with a trend towards upregulation vs WT, with no significant changes observed in *MMP9* but a trend towards increased *TIMP1* expression in DM_CKD vs WT as well as DM. These results suggest that combining diabetes and CKD worsened cardiac fibrosis through enhanced collagen deposition and selective ECM remodeling.

## Discussion

The main findings of our study in swine are that while DM alone substantially impacts cardiac hemodynamics, with elevated cardiac pre- and afterload provoking elevated coronary blood flow at rest which compromises coronary flow reserve, but also maladaptive cardiac remodeling with diastolic, systolic, and mitochondrial dysfunction, the addition of CKD causes no or little additional compromise on these parameters. However, the addition of CKD to DM did increase oxidative stress in LV myocardium and the coronary vasculature. These findings were substantiated by upregulation of similar oxidative stress-related pathways in myocardial samples from patients with HFpEF, DM, and CKD, suggesting that oxidative stress may play an important role in the pathophysiology of diabetic and uremic cardiomyopathy. Together, the parallel observations in swine and human myocardium emphasize that CKD amplifies DM-induced cardiac damage principally via increased oxidative stress and fibrotic remodeling, thereby qualifying these pathways as targets for early intervention in cardiorenal syndrome.

Diabetes exerts a multifaceted impact on the heart, driving metabolic alterations that result in reduced ATP production, increased generation of ROS, and altered contractile function, ultimately culminating in diabetic cardiomyopathy [26]. In our swine model, we observed that diabetes was the main driver of the changes in cardiac function, resulting in ventricular dilation and increased pressure, consistent with clinical data on patient populations with diabetes [54, 75]. It should, however, be noted that diabetic cardiomyopathy encompasses different phenotypes, including patients with preserved as well as midrange and reduced ejection fraction [41]. In a recent meta-analysis, diastolic dysfunction showed a prevalence of 43%, whereas systolic dysfunction was present in 6% of patients with DM [23], but the mechanisms underlying these divergent responses remain incompletely understood. Our swine model mimics this clinical diversity, but indicates that DM alone results in a volume overload, leading to LV dilation and a combination of impaired filling (E/A) and systolic dysfunction with the increase in systolic wall stress being due to the volume overload. Addition of CKD increased Ped despite a smaller increase in Ved, which resulted in an increase in diastolic wall stress, that likely contributed to the decrease in E/A in these animals. Interestingly, clinical data from the Zoppini et al. study showed that patients with the lowest glomerular filtration rate (GFR) exhibited the highest filling pressures, suggesting a complex interplay between diabetes and CKD that may influence cardiac remodeling [75].

CKD and diabetes can induce endothelial dysfunction, which in turn may cause functional coronary microvascular disease as evidenced by increased basal coronary flow and a reduction in coronary flow reserve in our swine. In our study, both DM and DM_CKD animals exhibited similar levels of elevated coronary flow as well as reduced coronary flow reserve. Along with this, we detected evidence of endothelial dysfunction in DM and DM_CKD, as indicated by reduced peNOS/eNOS ratios [60, 64]. This was accompanied by increased VEGF particularly in the DM_CKD group implying a compensatory and/or dysfunctional angiogenic response [52, 53]. In healthy hearts, coronary blood flow is tightly coupled to myocardial metabolism, enabling efficient (75–80%) utilization of the oxygen delivered through the coronary vasculature [61]. Furthermore, it has been suggested that mitochondrial function plays a seminal role in coupling myocardial blood flow to metabolism, while diabetes and obesity induce mitochondrial DNA damage and thereby impair coronary metabolic vasodilation [20]. In contrast, we observed an increase basal coronary blood flow, which is in accordance with observations in humans with diabetes and CKD [35, 43, 50], but also patients with HFpEF in general [1] and is likely to be explained by the heart’s heightened metabolic and oxygen demands imposed by elevated cardiac after- and/or preload [2]. Previous studies also reported altered substrate utilization and mitochondrial inefficiency as typical sequelae in diabetic myocardium [30, 35, 43, 50]. In fact, a metabolic shift from glucose towards more fatty acid oxidation and in particular, mitochondrial overload with fatty acids can cause mitochondrial uncoupling and damage, where uncoupling would demand more O_2_ consumption per unit of ATP production [8, 11, 14]. However, in our experiments, we observed no mitochondrial uncoupling (i.e., unchanged State 4 respiration), but reduced maximal respiratory capacity (i.e., reduced State 3 respiration) in response to any provided substrate (complex I or II, glutamate, fatty acids), rendering mitochondrial uncoupling per se unlikely to explain the increase in baseline coronary blood flow. Since mitochondria were isolated from whole myocardium, the changes in maximal respiratory capacity likely reflect changes in cardiomyocyte-derived mitochondria. Given that dysregulation of microvascular mitochondria has been shown to induce endothelial dysfunction, and thereby also influence cardiac metabolism and inflammation [21], future studies should specifically assess the impact of diabetes and CKD on these microvascular mitochondria.

Proteomic analysis of LV tissue revealed marked inflammatory and metabolic adaptations in both DM and DM_CKD groups. In particular, diabetes was associated with significant alterations in fatty acid metabolism, notably through increased expression of PPARα in the DM_CKD group. Activation of PPARα drives elevated free fatty acid (FFA) oxidation and utilization, initially serving as a compensatory response to heightened metabolic demand in the diabetic heart and also shown in other porcine models of cardiorenal syndrome [10, 17]. However, chronic overexpression of fatty acid metabolism has been shown to reduce Ca^2^⁺ uptake, promote LV hypertrophy, impair systolic function, and increase the cardiac injury markers, all of which contribute to long-term cardiac dysfunction and remodeling [2]. This imbalance between fatty acid uptake and oxidation can also lead to the accumulation of ROS, further exacerbating oxidative stress and structural changes in the myocardium [44]. We also detected increased lipid peroxidation in the proteomics which was supported by the increased 4-HNE adducts in the DM_CKD. In addition, our proteomic data indicated alteration of glutamate metabolism in diabetic animals which is linked to impaired insulin secretion, increased insulin resistance, and reduced synthesis of glutathione which is a key antioxidant critical for mitochondrial redox balance and cardiomyocyte protection [66, 71, 74]. PBMCs from coronary artery disease (CMD) patients with CKD expressed increased amounts of the enzyme GCLM (glutamate cysteine ligase modifier) [73], which was congruent with our finding of increased glutamate metabolism in the DM_CKD animals. Hence, increased GCLM in DM_CKD hearts likely reflects a compensatory upregulation aimed at replenishing glutathione and protecting mitochondrial and cellular integrity under conditions of oxidative stress and mitochondrial dysfunction [16, 70].

Mitochondrial dysfunction was further proven using mitochondrial respiration assays. Specifically, State 3 respiration, which is critical for ATP generation, was significantly impaired in the diabetic groups, while baseline (State 4) respiration remained unchanged. This pattern has been demonstrated in other models of swine with diabetes or CKM [2, 10, 15, 48] and may contribute to cardiac energy starvation, a factor characteristic for HF in general that may particularly impact maximal cardiac function during exertion [8, 38]. However, the metabolic inflexibility, as evidenced by reduced mitochondrial oxidation of fatty acids, pyruvate-malate, and glutamate-malate, was not further compounded by CKD in our study. It is important to note that the DM-CKD animals received more insulin, which may have reduced the impact of DM on the heart, and particularly on mitochondrial function. The human proteome data also pointed towards mitochondrial dysfunction in the HFpEF patients with DM and CKD.

Interestingly, despite oxidative stress on the tissue level, H₂O₂ emission from isolated mitochondria respiring on physiological substrates with physiological ADP concentrations was not increased in DM or DM_CKD compared to WT conditions. This does not rule out that in intact cardiac myocytes in the in vivo conditions, mitochondrial ROS could be elevated, since especially in HFpEF, increased pre- and afterload demand excessive ATP production in mitochondria, which oxidizes NADPH—normally required to regenerate glutathione and thereby detoxify H_2_O_2_—via the reverse mode of the mitochondrial transhydrogenase (NNT) [39, 45]. In fact, mice lacking a functional NNT are resistant against oxidative stress and HF remodeling in response to elevated cardiac afterload alone or induction of HFpEF by a high-fat diet and the vasopressor L-NAME [19, 36, 39, 45]. Therefore, mechano-energetic uncoupling imposed by increased hemodynamic load, which hampers mitochondrial ROS detoxification, rather than an increase of ROS production at the respiratory chain per se, as has been observed in large animal models with HF with *reduced* ejection fraction (HFrEF) [24] may underlie the increase in oxidative stress in HFpEF induced by DM and CKD. Such mitochondrial ROS can additionally hamper mitochondrial and cellular energetics by deteriorating respiratory chain function and inhibiting the activity of the creatine kinase as an important transmitter of the cellular energy [49, 72].

The presence of oxidative stress in DM_CKD tissue was further supported by our observation of decreased GPX3 levels as well as increased lipid peroxidation in LV tissue, which is consistent with data from mice models of CKD and GPX1 levels in patients with diabetic kidney disease [42, 76]. This was accompanied by increased levels of PRDX1 and SOD2 in the swine and SOD1 in the humans. Since SOD converts superoxide to H₂O₂, its upregulation can elevate H₂O₂ levels, which not only acts as a vasodilator—potentially explaining the increased basal coronary flow observed in diabetic and diabetic-CKD animals—but also leads to greater oxidative stress when GPX-mediated detoxification is compromised, resulting in increased tissue damage [44]. Mice studies on a CKD model combined with *Gpx3* knockout have shown LV dysfunction and increased platelet aggregation [42]. In addition, elevated 8-HDG and 4-HNE levels in our model further confirm increased ROS production beyond H₂O₂ accumulation, indicating widespread oxidative damage in diabetic CKD myocardium.

Finally, our proteomics study also revealed alterations in the extracellular remodeling proteins which can be triggered by the increased preload in combination with oxidative damage in the diabetic animals in the presence of CKD. Exacerbated oxidative stress can promote ECM remodeling in the LV tissues [27, 54, 59] as evidenced by increased collagen and MMP2 indicating fibrosis in the present study. MMP2 interacts with TGF-β signaling, a key driver of fibrosis and ECM deposition, further promoting fibrotic remodeling in cardiac tissue. Furthermore, there are studies showing matrix metalloproteinase-mediated loss of the endothelial glycocalyx, promoting endothelial dysfunction and collectively reflecting the complex interplay of oxidative damage, metabolic disturbances, and organ crosstalk driving cardiac fibrosis and dysfunction in the setting of diabetes and CKD [3].

### Future perspectives

The treatment landscape for patients with diabetes and CKD is rapidly evolving with SGLT2 inhibitors and GLP1 agonists*.* There is a growing recognition for the need for a common therapeutic regimen to target organ crosstalk to treat this cardiorenal feedback. Efforts to translate preclinical findings on anti-oxidative agents into clinical benefit have largely been unsuccessful, highlighting gaps in our mechanistic understanding of how metabolic, inflammatory, and oxidative pathways interact in the diabetes and cardiorenal syndrome. Future research must focus on elucidating these mechanistic subtleties, including the roles of mitochondrial dysfunction, endothelial signaling, and extracellular matrix remodeling, to identify novel therapeutic targets and refine combination strategies.

## Conclusion

**Fig. 10:**
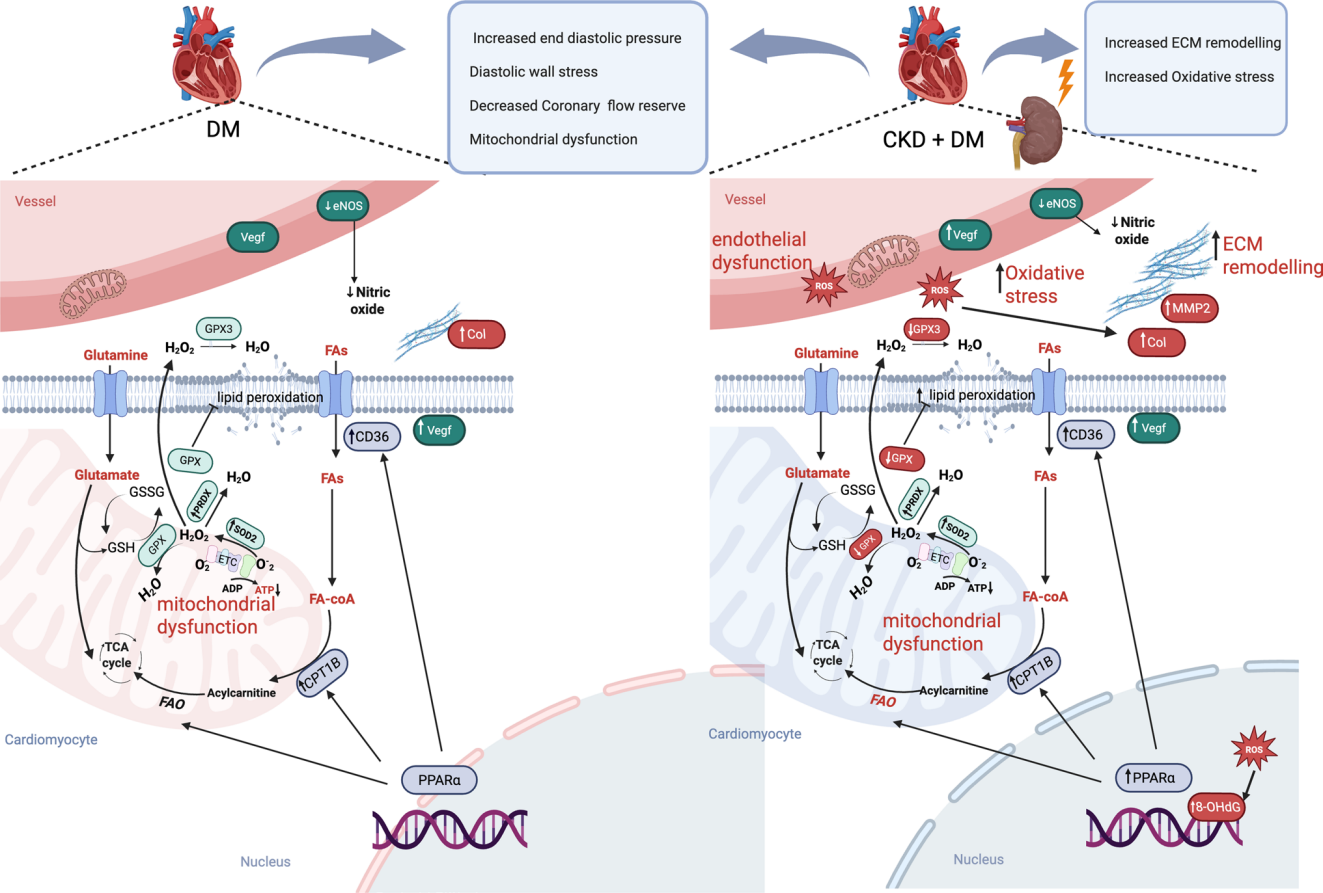
Schematic representation of cardiac metabolic and oxidative stress alterations in swine with diabetes and chronic kidney disease (CKD) increased vascular endothelial growth factor (VEGF) expression, and reduced endothelial nitric oxide synthase (eNOS) levels are observed in the DM_CKD swine. Cardiomyocytes exhibit elevated fatty acid oxidation, indicated by increased expression of peroxisome proliferator-activated receptor alpha (PPARα), carnitine palmitoyltransferase (CPT), and cluster of differentiation 36 (CD36), but show reduced ATP generation, suggesting mitochondrial uncoupling. Altered glutamate metabolism impairs synthesis of glutathione (GSH), a key antioxidant. Increased superoxide dismutase 2 (SOD2) activity raises H₂O₂ levels, collectively leading to enhanced reactive oxygen species (ROS) production. Reduced glutathione peroxidase (GPX) activity further diminishes glutathione-mediated inhibition of lipid peroxidation, exacerbating ROS accumulation. The cumulative effect of these metabolic and redox imbalances drives extracellular matrix (ECM) remodeling in the heart

Taken together, our data show that DM alone impairs endothelial NO signaling, deteriorates mitochondrial function, and imposes hemodynamic load on the LV that through mechano-energetic uncoupling may contribute to energetic deficit and oxidative stress, relevant drivers of HF development. While the addition of CKD does not further aggravate the above factors, it yet creates a more pro-oxidative environment by a further deterioration of the anti-oxidative capacity and further accentuates metabolic rewiring and ECM remodeling, that in concert may accelerate the progression of HF development in the longer term (Fig. 10).

Collectively, these findings highlight the potential for early identification and intervention to prevent the progression of diabetic cardiomyopathy in patients with concurrent CKD.

## Supplementary Information

Below is the link to the electronic supplementary material.

Supplementary file1 (XLSX 563 KB)

Supplementary file2 (TIFF 6596 KB)

Supplementary file3 (TIFF 6596 KB)

Supplementary file4 (TIFF 6596 KB)

Supplementary file5 (TIFF 6596 KB)

## Data Availability

All data are available in the online data supplement as well as from the PRIDE repository (proteomics, see also Methods section).

## References

[1] AbouEzzeddine OF, Kemp BJ, Borlaug BA, Mullan BP, Behfar A, Pislaru SV, Fudim M, Redfield MM, Chareonthaitawee P (2019) Myocardial energetics in heart failure with preserved ejection fraction. Circ Heart Fail 12:e006240. 10.1161/CIRCHEARTFAILURE.119.006240 31610726 10.1161/CIRCHEARTFAILURE.119.006240 PMC6863599

[2] Aksentijevic D, Sedej S, Fauconnier J, Paillard M, Abdellatif M, Streckfuss-Bömeke K, Ventura-Clapier R, van der Velden J, de Boer RA, Bertero E, Dudek J, Sequeira V, Maack C (2025) Mechano-energetic uncoupling in heart failure. Nat Rev Cardiol 22:773–797. 10.1038/s41569-025-01167-6 40544170 10.1038/s41569-025-01167-6

[3] Ali MM, Mahmoud AM, Le Master E, Levitan I, Phillips SA (2019) Role of matrix metalloproteinases and histone deacetylase in oxidative stress-induced degradation of the endothelial glycocalyx. Am J Physiol Heart Circ Physiol 316:H647–H663. 10.1152/ajpheart.00090.2018 30632766 10.1152/ajpheart.00090.2018 PMC6459309

[4] Ammar C, Gruber M, Csaba G, Zimmer R (2019) MS-EmpiRe utilizes peptide-level noise distributions for ultra-sensitive detection of differentially expressed proteins. Mol Cell Proteomics 18:1880–1892. 10.1074/mcp.RA119.001509 31235637 10.1074/mcp.RA119.001509 PMC6731086

[5] Baaten C, Vondenhoff S, Noels H (2023) Endothelial cell dysfunction and increased cardiovascular risk in patients with chronic kidney disease. Circ Res 132:970–992. 10.1161/CIRCRESAHA.123.321752 37053275 10.1161/CIRCRESAHA.123.321752 PMC10097498

[6] Backman M, Flenkenthaler F, Blutke A, Dahlhoff M, Ländström E, Renner S, Philippou-Massier J, Krebs S, Rathkolb B, Prehn C, Grzybek M, Coskun Ü, Rothe M, Adamski J, de Angelis MH, Wanke R, Fröhlich T, Arnold GJ, Blum H, Wolf E (2019) Multi-omics insights into functional alterations of the liver in insulin-deficient diabetes mellitus. Mol Metab 26:30–44. 10.1016/j.molmet.2019.05.011 31221621 10.1016/j.molmet.2019.05.011 PMC6667734

[7] Beikoghli Kalkhoran S, Basalay M, He Z, Golforoush P, Roper T, Caplin B, Salama AD, Davidson SM, Yellon DM (2024) Investigating the cause of cardiovascular dysfunction in chronic kidney disease: capillary rarefaction and inflammation may contribute to detrimental cardiovascular outcomes. Basic Res Cardiol 119:937–955. 10.1007/s00395-024-01086-6 39472324 10.1007/s00395-024-01086-6 PMC11628583

[8] Bertero E, Maack C (2018) Metabolic remodelling in heart failure. Nat Rev Cardiol 15:457–470. 10.1038/s41569-018-0044-6 29915254 10.1038/s41569-018-0044-6

[9] Braunwald E (2019) Diabetes, heart failure, and renal dysfunction: the vicious circles. Prog Cardiovasc Dis 62:298–302. 10.1016/j.pcad.2019.07.003 31377223 10.1016/j.pcad.2019.07.003

[10] Chade AR, Sitz R, Kelty TJ, McCarthy E, Tharp DL, Rector RS, Eirin A (2024) Chronic kidney disease and left ventricular diastolic dysfunction (CKD-LVDD) alter cardiac expression of mitochondria-related genes in swine. Transl Res 267:67–78. 10.1016/j.trsl.2023.12.004 38262578 10.1016/j.trsl.2023.12.004 PMC11001533

[11] Cortassa S, Sollott SJ, Aon MA (2017) Mitochondrial respiration and ROS emission during β-oxidation in the heart: an experimental-computational study. PLoS Comput Biol 13:1–23. 10.1371/journal.pcbi.1005588 10.1371/journal.pcbi.1005588 PMC5482492 28598967

[12] D’Oria R, Schipani R, Leonardini A, Natalicchio A, Perrini S, Cignarelli A, Laviola L, Giorgino F (2020) The role of oxidative stress in cardiac disease: from physiological response to injury factor. Oxid Med Cell Longev. 10.1155/2020/5732956 32509147 10.1155/2020/5732956 PMC7244977

[13] Debard C, Laville M, Berbe V, Loizon E, Guillet C, Morio-Liondore B, Boirie Y, Vidal H (2004) Expression of key genes of fatty acid oxidation, including adiponectin receptors, in skeletal muscle of type 2 diabetic patients. Diabetologia 47:917–925. 10.1007/s00125-004-1394-7 15127202 10.1007/s00125-004-1394-7

[14] Echtay KS, Roussel D, St-Pierre J, Jekabsons MB, Cadenas S, Stuart JA, Harper JA, Roebuck SJ, Morrison A, Pickering S, Clapham JC, Brand MD (2002) Superoxide activates mitochondrial uncoupling proteins. Nature 415:96–99. 10.1038/415096a 11780125 10.1038/415096a

[15] Eirin A, Ebrahimi B, Kwon SH, Fiala JA, Williams BJ, Woollard JR, He Q, Gupta RC, Sabbah HN, Prakash YS, Textor SC, Lerman A, Lerman LO (2016) Restoration of mitochondrial cardiolipin attenuates cardiac damage in swine renovascular hypertension. J Am Heart Assoc 5:1–19. 10.1161/JAHA.115.003118 10.1161/JAHA.115.003118 PMC4937260 27247333

[16] Fassett RG, Robertson IK, Ball MJ, Geraghty DP, Coombes JS (2015) Effects of atorvastatin on oxidative stress in chronic kidney disease. Nephrology (Carlton) 20:697–705. 10.1111/nep.12502 25959591 10.1111/nep.12502

[17] Finck BN, Lehman JJ, Leone TC, Welch MJ, Bennett MJ, Kovacs A, Han X, Gross RW, Kozak R, Lopaschuk GD, Kelly DP (2002) The cardiac phenotype induced by PPARalpha overexpression mimics that caused by diabetes mellitus. J Clin Invest 109:121–130. 10.1172/JCI14080 11781357 10.1172/JCI14080 PMC150824

[18] Francis A, Harhay MN, Ong ACM, Tummalapalli SL, Ortiz A, Fogo AB, Fliser D, Roy-Chaudhury P, Fontana M, Nangaku M, Wanner C, Malik C, Hradsky A, Adu D, Bavanandan S, Cusumano A, Sola L, Ulasi I, Jha V (2024) Chronic kidney disease and the global public health agenda: an international consensus. Nat Rev Nephrol 20:473–485. 10.1038/s41581-024-00820-6 38570631 10.1038/s41581-024-00820-6

[19] Garcia-Menendez L, Karamanlidis G, Kolwicz S, Tian R (2013) Substrain specific response to cardiac pressure overload in C57BL/6 mice. Am J Physiol Heart Circ Physiol 305:H397-402. 10.1152/ajpheart.00088.2013 23709599 10.1152/ajpheart.00088.2013 PMC3742875

[20] Guarini G, Kiyooka T, Ohanyan V, Pung YF, Marzilli M, Chen YR, Chen CL, Kang PT, Hardwick JP, Kolz CL, Yin L, Wilson GL, Shokolenko I, Dobson JG Jr, Fenton R, Chilian WM (2016) Impaired coronary metabolic dilation in the metabolic syndrome is linked to mitochondrial dysfunction and mitochondrial DNA damage. Basic Res Cardiol 111:29. 10.1007/s00395-016-0547-4 27040114 10.1007/s00395-016-0547-4 PMC5542063

[21] Gutierrez-Huerta CA, Quiroz-Delfi G, Faleel FDM, Beyer AM (2025) Impaired endothelial function contributes to cardiac dysfunction: role of mitochondrial dynamics. Am J Physiol Heart Circ Physiol 328:H29–H36. 10.1152/ajpheart.00531.2024 39560973 10.1152/ajpheart.00531.2024 PMC12147231

[22] Heusch G, Andreadou I, Bell R, Bertero E, Botker H-E, Davidson SM, Downey J, Eaton P, Ferdinandy P, Gersh BJ, Giacca M, Hausenloy DJ, Ibanez B, Krieg T, Maack C, Schulz R, Sellke F, Shah AM, Thiele H, Yellon DM, Di Lisa F (2023) Health position paper and redox perspectives on reactive oxygen species as signals and targets of cardioprotection. Redox Biol 67:102894. 10.1016/j.redox.2023.102894 37839355 10.1016/j.redox.2023.102894 PMC10590874

[23] Hoek AG, Dal Canto E, Wenker E, Bindraban N, Handoko ML, Elders PJM, Beulens JWJ (2024) Epidemiology of heart failure in diabetes: a disease in disguise. Diabetologia 67:574–601. 10.1007/s00125-023-06068-2 38334818 10.1007/s00125-023-06068-2 PMC10904471

[24] Ide T, Tsutsui H, Kinugawa S, Utsumi H, Kang D, Hattori N, Uchida K, Arimura K, Egashira K, Takeshita A (1999) Mitochondrial electron transport complex I is a potential source of oxygen free radicals in the failing myocardium. Circ Res 85:357–363. 10.1161/01.res.85.4.357 10455064 10.1161/01.res.85.4.357

[25] Jani VP, Yoo EJ, Binek A, Guo A, Kim JS, Aguilan J, Keykhaei M, Jenkin SR, Sidoli S, Sharma K, Van Eyk JE, Kass DA, Hahn VS (2025) Myocardial proteome in human heart failure with preserved ejection fraction. J Am Heart Assoc 14:e038945–e038945. 10.1161/JAHA.124.038945 40079330 10.1161/JAHA.124.038945 PMC12132713

[26] Jia G, Hill MA, Sowers JR (2018) Diabetic cardiomyopathy: an update of mechanisms contributing to this clinical entity. Circ Res 122:624–638. 10.1161/CIRCRESAHA.117.311586 29449364 10.1161/CIRCRESAHA.117.311586 PMC5819359

[27] Kaesler N, Cheng M, Nagai J, O’Sullivan J, Peisker F, Bindels EMJ, Babler A, Moellmann J, Droste P, Franciosa G, Dugourd A, Saez-Rodriguez J, Neuss S, Lehrke M, Boor P, Goettsch C, Olsen JV, Speer T, Lu T-S, Lim K, Floege J, Denby L, Costa I, Kramann R (2025) Mapping cardiac remodeling in chronic kidney disease. Sci Adv 9:eadj4846-eadj4846. 10.1126/sciadv.adj4846 10.1126/sciadv.adj4846 PMC10672229 38000021

[28] Kundu S, Gairola S, Verma S, Mugale MN, Sahu BD (2024) Chronic kidney disease activates the HDAC6-inflammatory axis in the heart and contributes to myocardial remodeling in mice: inhibition of HDAC6 alleviates chronic kidney disease-induced myocardial remodeling. Basic Res Cardiol 119:831–852. 10.1007/s00395-024-01056-y 38771318 10.1007/s00395-024-01056-y

[29] Li Y, Liu Y, Liu S, Gao M, Wang W, Chen K, Huang L, Liu Y (2023) Diabetic vascular diseases: molecular mechanisms and therapeutic strategies. Signal Transduct Target Ther. 10.1038/s41392-023-01400-z 37037849 10.1038/s41392-023-01400-z PMC10086073

[30] Liberale L, Duncker DJ, Hausenloy DJ, Kraler S, Bøtker HE, Podesser BK, Heusch G, Kleinbongard P (2025) Vascular (dys)function in the failing heart. Nat Rev Cardiol 22:728–750. 10.1038/s41569-025-01163-w 40544172 10.1038/s41569-025-01163-w

[31] Ljubojević-Holzer S, Kraler S, Djalinac N, Abdellatif M, Voglhuber J, Schipke J, Schmidt M, Kling K-M, Franke GT, Herbst V, Zirlik A, von Lewinski D, Scherr D, Rainer PP, Kohlhaas M, Nickel A, Mühlfeld C, Maack C, Sedej S (2022) Loss of autophagy protein ATG5 impairs cardiac capacity in mice and humans through diminishing mitochondrial abundance and disrupting Ca2+ cycling. Cardiovasc Res 118:1492–1505. 10.1093/cvr/cvab112 33752242 10.1093/cvr/cvab112 PMC9074988

[32] Maack C, Lehrke M, Backs J, Heinzel FR, Hulot JS, Marx N, Paulus WJ, Rossignol P, Taegtmeyer H, Bauersachs J, Bayes-Genis A, Brutsaert D, Bugger H, Clarke K, Cosentino F, De Keulenaer G, Dei Cas A, Gonzalez A, Huelsmann M, Iaccarino G, Lunde IG, Lyon AR, Pollesello P, Rena G, Riksen NP, Rosano G, Staels B, van Laake LW, Wanner C, Farmakis D, Filippatos G, Ruschitzka F, Seferovic P, de Boer RA, Heymans S (2018) Heart failure and diabetes: metabolic alterations and therapeutic interventions: a state-of-the-art review from the Translational Research Committee of the Heart Failure Association-European Society of Cardiology. Eur Heart J 39:4243–4254. 10.1093/eurheartj/ehy596 30295797 10.1093/eurheartj/ehy596 PMC6302261

[33] Maack C, Tardiff JC (2022) Targeted therapies for cardiac diseases. Nat Rev Cardiol 19:343–344. 10.1038/s41569-022-00704-x 35468998 10.1038/s41569-022-00704-x

[34] Mela L, Seitz S (1979) Isolation of mitochondria with emphasis on heart mitochondria from small amounts of tissue. Methods Enzymol 55:39–46. 10.1016/0076-6879(79)55006-x 459851 10.1016/0076-6879(79)55006-x

[35] Mohandas R, Segal MS, Huo T, Handberg EM, Petersen JW, Johnson BD, Sopko G, Bairey Merz CN, Pepine CJ (2015) Renal function and coronary microvascular dysfunction in women with symptoms/signs of ischemia. PLoS ONE 10:e0125374-e0125374. 10.1371/journal.pone.0125374 25951606 10.1371/journal.pone.0125374 PMC4423851

[36] Muller M, Bischof C, Kapries T, Wollnitza S, Liechty C, Geissen S, Schubert T, Opacic D, Gercek M, Fortmeier V, Dumitrescu D, Schlomann U, Sydykov A, Petrovic A, Gnatzy-Feik L, Milting H, Schermuly RT, Friedrichs K, Rudolph V, Klinke A (2022) Right heart failure in mice upon pressure overload is promoted by mitochondrial oxidative stress. JACC Basic Transl Sci 7:658–677. 10.1016/j.jacbts.2022.02.018 35958691 10.1016/j.jacbts.2022.02.018 PMC9357563

[37] Ndumele CE, Rangaswami J, Chow SL, Neeland IJ, Tuttle KR, Khan SS, Coresh J, Mathew RO, Baker-Smith CM, Carnethon MR, Despres JP, Ho JE, Joseph JJ, Kernan WN, Khera A, Kosiborod MN, Lekavich CL, Lewis EF, Lo KB, Ozkan B, Palaniappan LP, Patel SS, Pencina MJ, Powell-Wiley TM, Sperling LS, Virani SS, Wright JT, Rajgopal Singh R, Elkind MSV (2023) Cardiovascular-kidney-metabolic health: a presidential advisory from the American Heart Association. Circulation 148:1606–1635. 10.1161/CIR.0000000000001184 37807924 10.1161/CIR.0000000000001184

[38] Neubauer S (2007) The failing heart–an engine out of fuel. N Engl J Med 356:1140–1151. 10.1056/NEJMra063052 17360992 10.1056/NEJMra063052

[39] Nickel AG, Von Hardenberg A, Hohl M, Löffler JR, Kohlhaas M, Becker J, Reil JC, Kazakov A, Bonnekoh J, Stadelmaier M, Puhl SL, Wagner M, Bogeski I, Cortassa S, Kappl R, Pasieka B, Lafontaine M, Lancaster CRD, Blacker TS, Hall AR, Duchen MR, Kästner L, Lipp P, Zeller T, Müller C, Knopp A, Laufs U, Böhm M, Hoth M, Maack C (2015) Reversal of mitochondrial transhydrogenase causes oxidative stress in heart failure. Cell Metab 22:472–484. 10.1016/j.cmet.2015.07.008 26256392 10.1016/j.cmet.2015.07.008

[40] Noels H, van der Vorst EPC, Rubin S, Emmett A, Marx N, Tomaszewski M, Jankowski J (2025) Renal-cardiac crosstalk in the pathogenesis and progression of heart failure. Circ Res 136:1306–1334. 10.1161/CIRCRESAHA.124.325488 40403103 10.1161/CIRCRESAHA.124.325488 PMC12105978

[41] Packer M (2021) Differential pathophysiological mechanisms in heart failure with a reduced or preserved ejection fraction in diabetes. JACC Heart failure 9:535–549. 10.1016/j.jchf.2021.05.019 34325884 10.1016/j.jchf.2021.05.019

[42] Pang P, Abbott M, Abdi M, Fucci QA, Chauhan N, Mistri M, Proctor B, Chin M, Wang B, Yin W, Lu TS, Halim A, Lim K, Handy DE, Loscalzo J, Siedlecki AM (2018) Pre-clinical model of severe glutathione peroxidase-3 deficiency and chronic kidney disease results in coronary artery thrombosis and depressed left ventricular function. Nephrol Dial Transplant 33:923–934. 10.1093/ndt/gfx304 29244159 10.1093/ndt/gfx304 PMC5982720

[43] Park S, Lee SH, Shin D, Hong D, Joh HS, Choi KH, Kim HK, Ha SJ, Park TK, Yang JH, Song YB, Hahn J-Y, Choi S-H, Gwon H-C, Lee JM (2023) Prognostic impact of coronary flow reserve in patients with CKD. Kidney Int Rep 8:64–74. 10.1016/j.ekir.2022.10.003 36644355 10.1016/j.ekir.2022.10.003 PMC9832048

[44] Peoples JN, Saraf A, Ghazal N, Pham TT, Kwong JQ (2019) Mitochondrial dysfunction and oxidative stress in heart disease. Exp Mol Med. 10.1038/s12276-019-0355-7 31857574 10.1038/s12276-019-0355-7 PMC6923355

[45] Pepin ME, Konrad PJM, Nazir S, Bazgir F, Maack C, Nickel A, Gorman JM, Hohl M, Schreiter F, Dewenter M, de Britto Chaves Filho A, Schulze A, Karlstaedt A, Frey N, Seidman CE, Seidman JG, Backs J (2025) Mitochondrial NNT promotes diastolic dysfunction in cardiometabolic HFpEF. Circ Res 136:1564–1578. 10.1161/CIRCRESAHA.125.32615440340422 10.1161/CIRCRESAHA.125.326154

[46] Perez-Riverol Y, Bai J, Bandla C, Garcia-Seisdedos D, Hewapathirana S, Kamatchinathan S, Kundu DJ, Prakash A, Frericks-Zipper A, Eisenacher M, Walzer M, Wang S, Brazma A, Vizcaino JA (2022) The PRIDE database resources in 2022: a hub for mass spectrometry-based proteomics evidences. Nucleic Acids Res 50:D543–D552. 10.1093/nar/gkab1038 34723319 10.1093/nar/gkab1038 PMC8728295

[47] Podkowinska A, Formanowicz D (2020) Chronic kidney disease as oxidative stress- and inflammatory-mediated cardiovascular disease. Antioxidants (Basel). 10.3390/antiox9080752 32823917 10.3390/antiox9080752 PMC7463588

[48] Polson SM, Thornburg JP, McNair BD, Cook CZ, Straight EA, Fontana KC, Hoopes CR, Nair S, Bruns DR (2024) Right ventricular dysfunction in preclinical models of type I and type II diabetes. Can J Physiol Pharmacol 103:86–97. 10.1139/cjpp-2024-0195 39693609 10.1139/cjpp-2024-0195

[49] Prag HA, Murphy MP, Krieg T (2023) Preventing mitochondrial reverse electron transport as a strategy for cardioprotection. Basic Res Cardiol 118:34. 10.1007/s00395-023-01002-4 37639068 10.1007/s00395-023-01002-4 PMC10462584

[50] Ragosta M, Samady H, Isaacs RB, Gimple LW, Sarembock IJ, Powers ER (2004) Coronary flow reserve abnormalities in patients with diabetes mellitus who have end-stage renal disease and normal epicardial coronary arteries. Am Heart J 147:1017–1023. 10.1016/j.ahj.2003.07.029 15199350 10.1016/j.ahj.2003.07.029

[51] Renner S, Braun-Reichhart C, Blutke A, Herbach N, Emrich D, Streckel E, Wünsch A, Kessler B, Kurome M, Bähr A, Klymiuk N, Krebs S, Puk O, Nagashima H, Graw J, Blum H, Wanke R, Wolf E (2013) Permanent neonatal diabetes in INS(C94Y) transgenic pigs. Diabetes 62:1505–1511. 10.2337/db12-1065 23274907 10.2337/db12-1065 PMC3636654

[52] Ricciardi CA, Gnudi L (2021) Vascular growth factors as potential new treatment in cardiorenal syndrome in diabetes. Eur J Clin Invest 51:1–11. 10.1111/eci.13579 10.1111/eci.13579 33942293

[53] Schrijvers BF, Flyvbjerg A, De Vriese AS (2004) The role of vascular endothelial growth factor (VEGF) in renal pathophysiology. Kidney Int 65:2003–2017. 10.1111/j.1523-1755.2004.00621.x 15149314 10.1111/j.1523-1755.2004.00621.x

[54] Sen P, Hamers J, Sittig T, Shashikadze B, d’Ambrosio L, Stöckl JB, Bierschenk S, Zhang H, d’Alessio C, Zandbergen LM, Pauly V, Clauss S, Wolf E, Dendorfer A, Fröhlich T, Merkus D (2024) Oxidative stress initiates hemodynamic change in CKD-induced heart disease. Basic Res Cardiol 119:957–971. 10.1007/s00395-024-01085-7 39404904 10.1007/s00395-024-01085-7 PMC11628585

[55] Soppert J, Frisch J, Wirth J, Hemmers C, Boor P, Kramann R, Vondenhoff S, Moellmann J, Lehrke M, Hohl M, van der Vorst EPC, Werner C, Speer T, Maack C, Marx N, Jankowski J, Roma LP, Noels H (2022) A systematic review and meta-analysis of murine models of uremic cardiomyopathy. Kidney Int 101:256–273. 10.1016/j.kint.2021.10.025 34774555 10.1016/j.kint.2021.10.025

[56] Sorop O, Heinonen I, van Kranenburg M, van de Wouw J, de Beer VJ, Nguyen ITN, Octavia Y, van Duin RWB, Stam K, van Geuns R-J, Wielopolski PA, Krestin GP, van den Meiracker AH, Verjans R, van Bilsen M, Danser AHJ, Paulus WJ, Cheng C, Linke WA, Joles JA, Verhaar MC, van der Velden J, Merkus D, Duncker DJ (2018) Multiple common comorbidities produce left ventricular diastolic dysfunction associated with coronary microvascular dysfunction, oxidative stress, and myocardial stiffening. Cardiovasc Res 114:954–964. 10.1093/cvr/cvy038 29432575 10.1093/cvr/cvy038 PMC5967461

[57] Streng KW, Nauta JF, Hillege HL, Anker SD, Cleland JG, Dickstein K, Filippatos G, Lang CC, Metra M, Ng LL, Ponikowski P, Samani NJ, van Veldhuisen DJ, Zwinderman AH, Zannad F, Damman K, van der Meer P, Voors AA (2018) Non-cardiac comorbidities in heart failure with reduced, mid-range and preserved ejection fraction. Int J Cardiol 271:132–139. 10.1016/j.ijcard.2018.04.001 30482453 10.1016/j.ijcard.2018.04.001

[58] Supek F, Bošnjak M, Škunca N, Šmuc T (2011) REVIGO summarizes and visualizes long lists of gene ontology terms. PLoS ONE 6:e21800. 10.1371/journal.pone.0021800 21789182 10.1371/journal.pone.0021800 PMC3138752

[59] Sygitowicz G, Maciejak-Jastrzebska A, Sitkiewicz D (2021) A review of the molecular mechanisms underlying cardiac fibrosis and atrial fibrillation. J Clin Med. 10.3390/jcm10194430 34640448 10.3390/jcm10194430 PMC8509789

[60] Tran N, Garcia T, Aniqa M, Ali S, Ally A, Nauli SM (2022) Endothelial nitric oxide synthase (eNOS) and the cardiovascular system: in physiology and in disease states. Am J Biomed Sci Res 15:153–177 35072089 PMC8774925

[61] Tune JD, Goodwill AG, Kiel AM, Baker HE, Bender SB, Merkus D, Duncker DJ (2020) Disentangling the Gordian knot of local metabolic control of coronary blood flow. Am J Physiol Heart Circ Physiol 318:H11–H24. 10.1152/ajpheart.00325.2019 31702972 10.1152/ajpheart.00325.2019 PMC7199237

[62] van de Wouw J, Broekhuizen M, Sorop O, Joles JA, Verhaar MC, Duncker DJ, Danser AHJ, Merkus D (2019) Chronic kidney disease as a risk factor for heart failure with preserved ejection fraction: a focus on microcirculatory factors and therapeutic targets. Front Physiol 10:1108. 10.3389/fphys.2019.01108 31551803 10.3389/fphys.2019.01108 PMC6737277

[63] van de Wouw J, Sorop O, van Drie RWA, van Duin RWB, Nguyen ITN, Joles JA, Verhaar MC, Merkus D, Duncker DJ (2020) Perturbations in myocardial perfusion and oxygen balance in swine with multiple risk factors: a novel model of ischemia and no obstructive coronary artery disease. Basic Res Cardiol 115:21. 10.1007/s00395-020-0778-2 32100119 10.1007/s00395-020-0778-2 PMC7042191

[64] van de Wouw J, Steenhorst JJ, Sorop O, van Drie RWA, Wielopolski PA, Kleinjan A, Hirsch A, Duncker DJ, Merkus D (2021) Impaired pulmonary vasomotor control in exercising swine with multiple comorbidities. Basic Res Cardiol 116:51. 10.1007/s00395-021-00891-7 34510273 10.1007/s00395-021-00891-7 PMC8435524

[65] van Drie RWA, van de Wouw J, Zandbergen LM, Dehairs J, Swinnen JV, Mulder MT, Verhaar MC, MaassenVanDenBrink A, Duncker DJ, Sorop O, Merkus D (2024) Vasodilator reactive oxygen species ameliorate perturbed myocardial oxygen delivery in exercising swine with multiple comorbidities. Basic Res Cardiol 119:869–887. 10.1007/s00395-024-01055-z 38796544 10.1007/s00395-024-01055-z PMC11461570

[66] Watanabe K, Nagao M, Toh R, Irino Y, Shinohara M, Iino T, Yoshikawa S, Tanaka H, Satomi-Kobayashi S, Ishida T, Hirata K-i (2021) Critical role of glutamine metabolism in cardiomyocytes under oxidative stress. Biochem Biophys Res Commun 534:687–693. 10.1016/j.bbrc.2020.11.018 33213841 10.1016/j.bbrc.2020.11.018

[67] Wei A-C, Aon MA, O’Rourke B, Winslow RL, Cortassa S (2011) Mitochondrial energetics, pH regulation, and ion dynamics: a computational-experimental approach. Biophys J 100:2894–2903. 10.1016/j.bpj.2011.05.027 21689522 10.1016/j.bpj.2011.05.027 PMC3123977

[68] Weissman D, Maack C (2021) Redox signaling in heart failure and therapeutic implications. Free Radic Biol Med 171:345–364. 10.1016/j.freeradbiomed.2021.05.013 34019933 10.1016/j.freeradbiomed.2021.05.013

[69] Wollenhaupt J, Frisch J, Harlacher E, Wong DWL, Jin H, Schulte C, Vondenhoff S, Moellmann J, Klinkhammer BM, Zhang L, Baleanu-Curaj A, Liehn EA, Speer T, Kazakov A, Werner C, van der Vorst EPC, Selejan SR, Hohl M, Bohm M, Kramann R, Biessen EAL, Lehrke M, Marx N, Jankowski J, Maack C, Boor P, Prates Roma L, Noels H (2022) Pro-oxidative priming but maintained cardiac function in a broad spectrum of murine models of chronic kidney disease. Redox Biol 56:102459. 10.1016/j.redox.2022.102459 36099852 10.1016/j.redox.2022.102459 PMC9482130

[70] Wong FN, Tan JAMA, Keng TC, Ng KP, Chua KH, Kuppusamy UR (2016) Association between plasma soluble RAGE and renal function is unaffected by medication usage and enzymatic antioxidants in chronic kidney disease with type 2 diabetes. Clin Chim Acta 453:56–61. 10.1016/j.cca.2015.12.002 26657980 10.1016/j.cca.2015.12.002

[71] Xing X, Sun Q, Wang R, Wang Y, Wang R (2024) Impacts of glutamate, an exercise-responsive metabolite on insulin signaling. Life Sci 341:122471-122471. 10.1016/j.lfs.2024.122471 38301875 10.1016/j.lfs.2024.122471

[72] Xu A, Weissman D, Ermer KJ, Bertero E, Federspiel JM, Stadler F, Grunler E, Tangos M, Zervou S, Waddingham MT, Pearson JT, Reil JC, Scholtz S, Dudek J, Kohlhaas M, Nickel AG, Carrier L, Eschenhagen T, Michels M, Dos Remedios C, Lal S, Prates Roma L, Hamdani N, Kuster D, Falcao-Pires I, Johnson CN, Lygate CA, van der Velden J, Maack C, Sequeira V (2025) Hypercontractility and oxidative stress drive creatine kinase dysfunction in hypertrophic cardiomyopathy. Circulation. 10.1161/CIRCULATIONAHA.125.074120 41111389 10.1161/CIRCULATIONAHA.125.074120 PMC13227915

[73] Yang B, Yang X, Sun H, Cheng M, Jin J, Wu Y, An Q, Yan K, Zhang S, Bai Y, Xu J (2025) Identification and validation of inflammatory response genes linking chronic kidney disease with coronary artery disease based on bioinformatics and machine learning. Sci Rep 15:1–15. 10.1038/s41598-025-03622-3 40451929 10.1038/s41598-025-03622-3 PMC12127440

[74] Yoo HC, Yu YC, Sung Y, Han JM (2020) Glutamine reliance in cell metabolism. Exp Mol Med 52:1496–1516. 10.1038/s12276-020-00504-8 32943735 10.1038/s12276-020-00504-8 PMC8080614

[75] Zoppini G, Bergamini C, Bonapace S, Trombetta M, Mantovani A, Toffalini A, Lanzoni L, Bertolini L, Zenari L, Bonora E, Targher G, Rossi A (2018) Left ventricular chamber dilation and filling pressure may help to categorise patients with type 2 diabetes. BMJ Open Diabetes Res Care 6:1–7. 10.1136/bmjdrc-2018-000529 10.1136/bmjdrc-2018-000529 PMC6014226 29942525

[76] Zou Z, Ren T, Li Y, Zeng Q, Wang X, Teng J, Xu J, Jia P, Ding X (2023) The association between serum glutathione peroxidase-3 concentration and risk of acute kidney injury after cardiac surgery: a nested case-control study. Am J Cardiol 209:29–35. 10.1016/j.amjcard.2023.08.141 37839462 10.1016/j.amjcard.2023.08.141

