# Supplementary figures and images for: From kidney injury to cardiac dysfunction: the central role of oxidative stress in diabetes and CKD
Source: Basic Res Cardiol. 2025 Dec 19;121(1):93–112. doi: 10.1007/s00395-025-01153-6 (PMC12804299; doi:10.1007/s00395-025-01153-6)

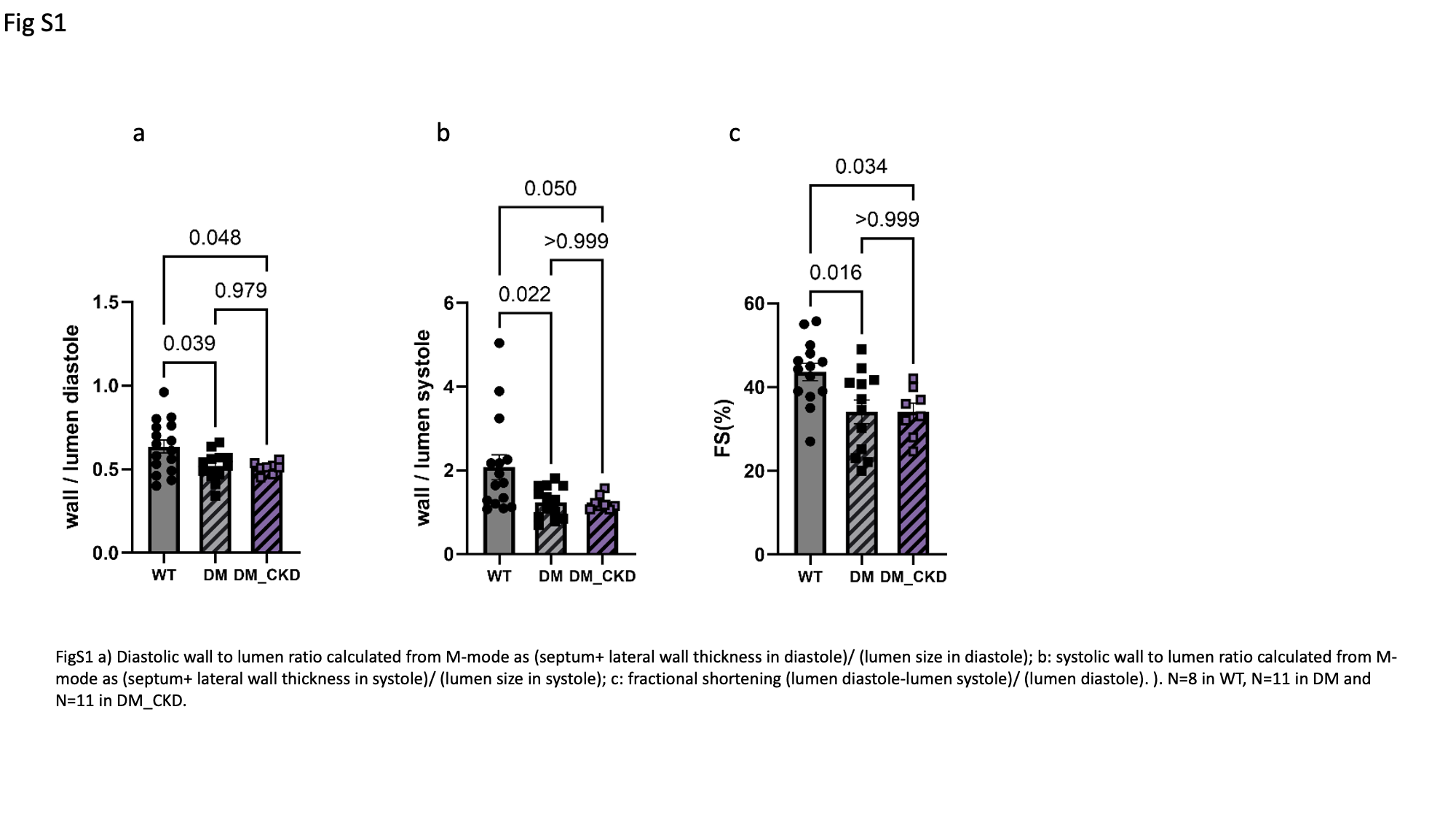

Supplement: Supplementary file 2 — Supplementary file2 (TIFF 6596 KB) [file 395_2025_1153_MOESM2_ESM.tiff]

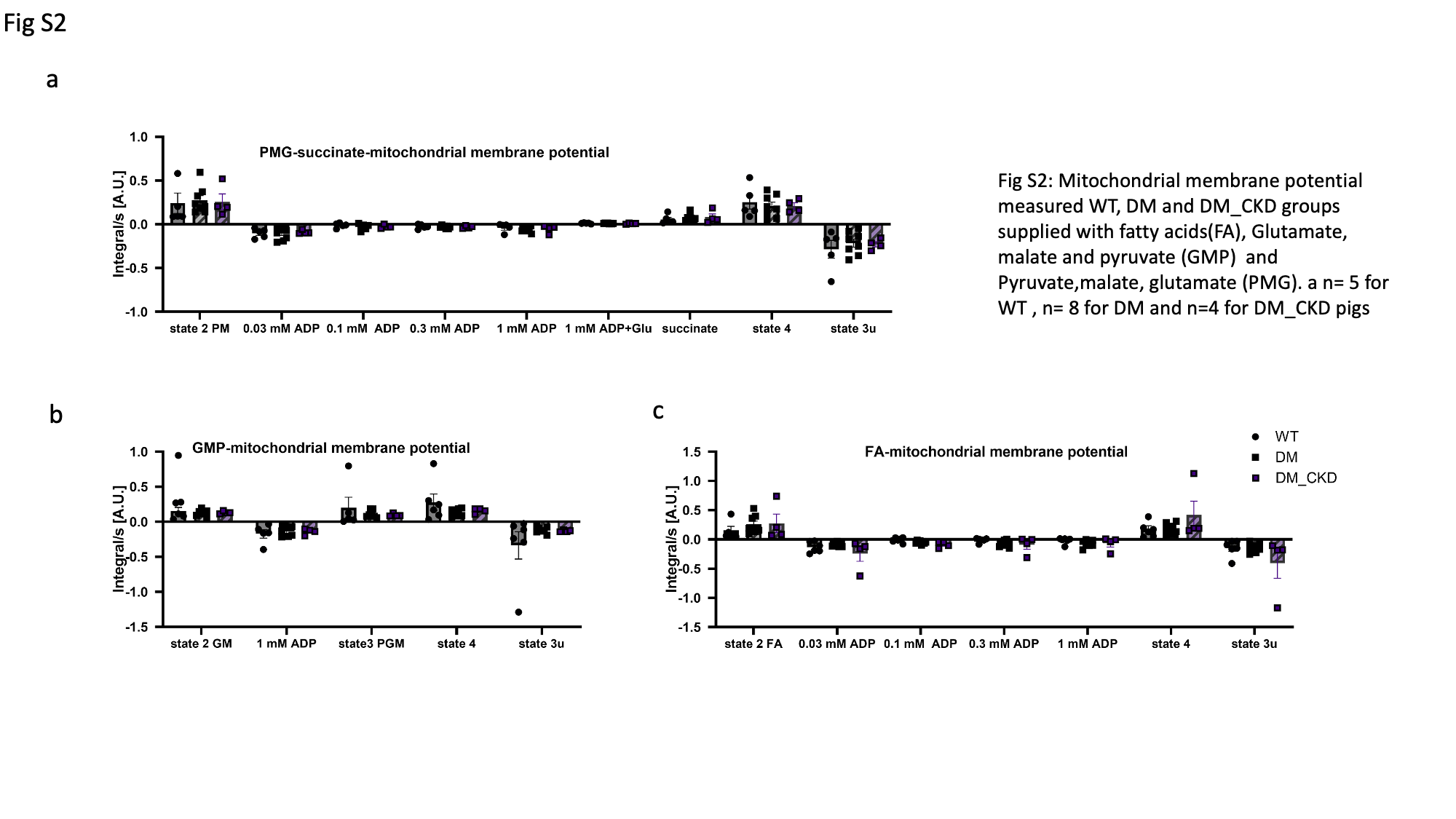

Supplement: Supplementary file 3 — Supplementary file3 (TIFF 6596 KB) [file 395_2025_1153_MOESM3_ESM.tiff]

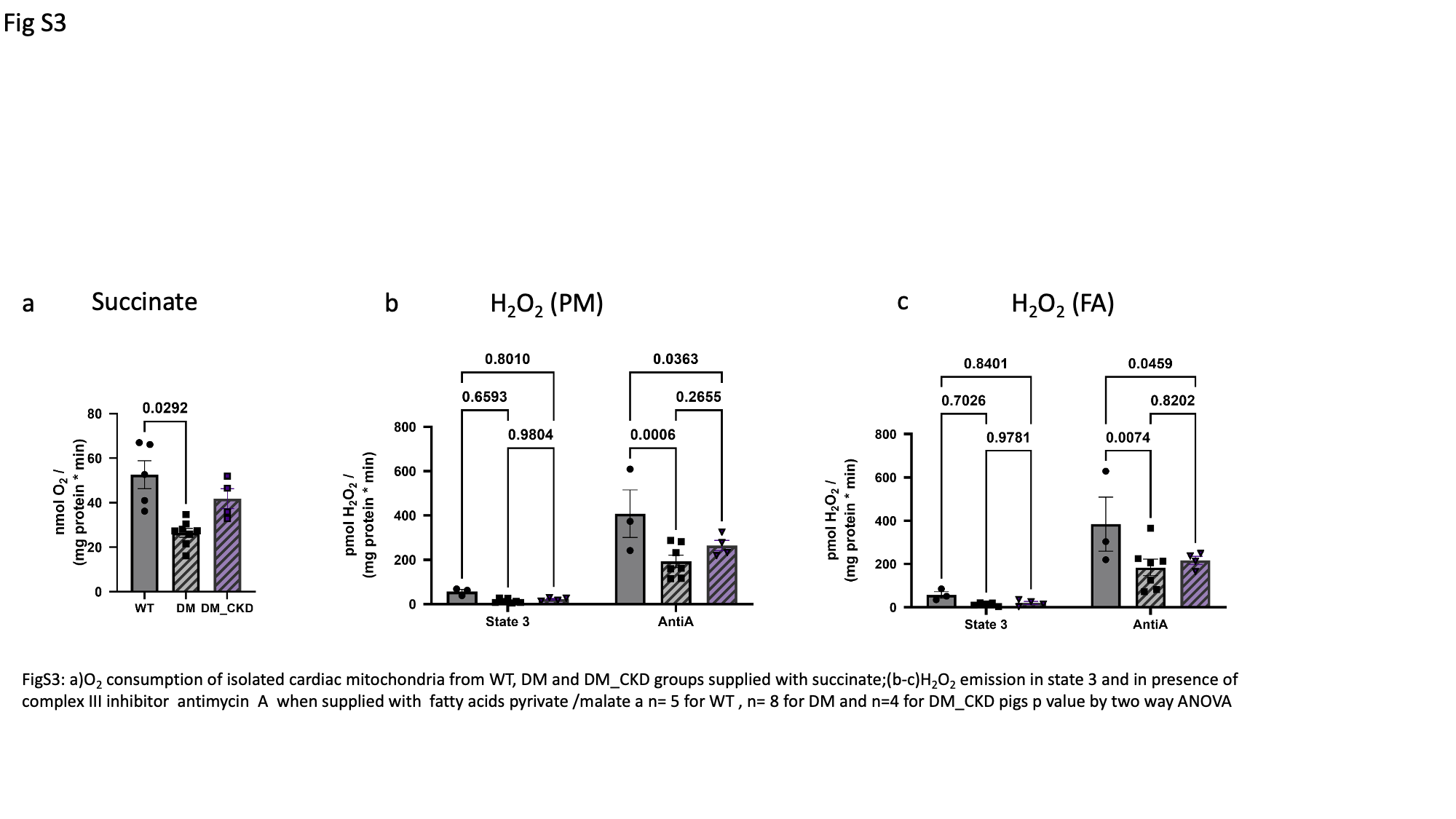

Supplement: Supplementary file 4 — Supplementary file4 (TIFF 6596 KB) [file 395_2025_1153_MOESM4_ESM.tiff]

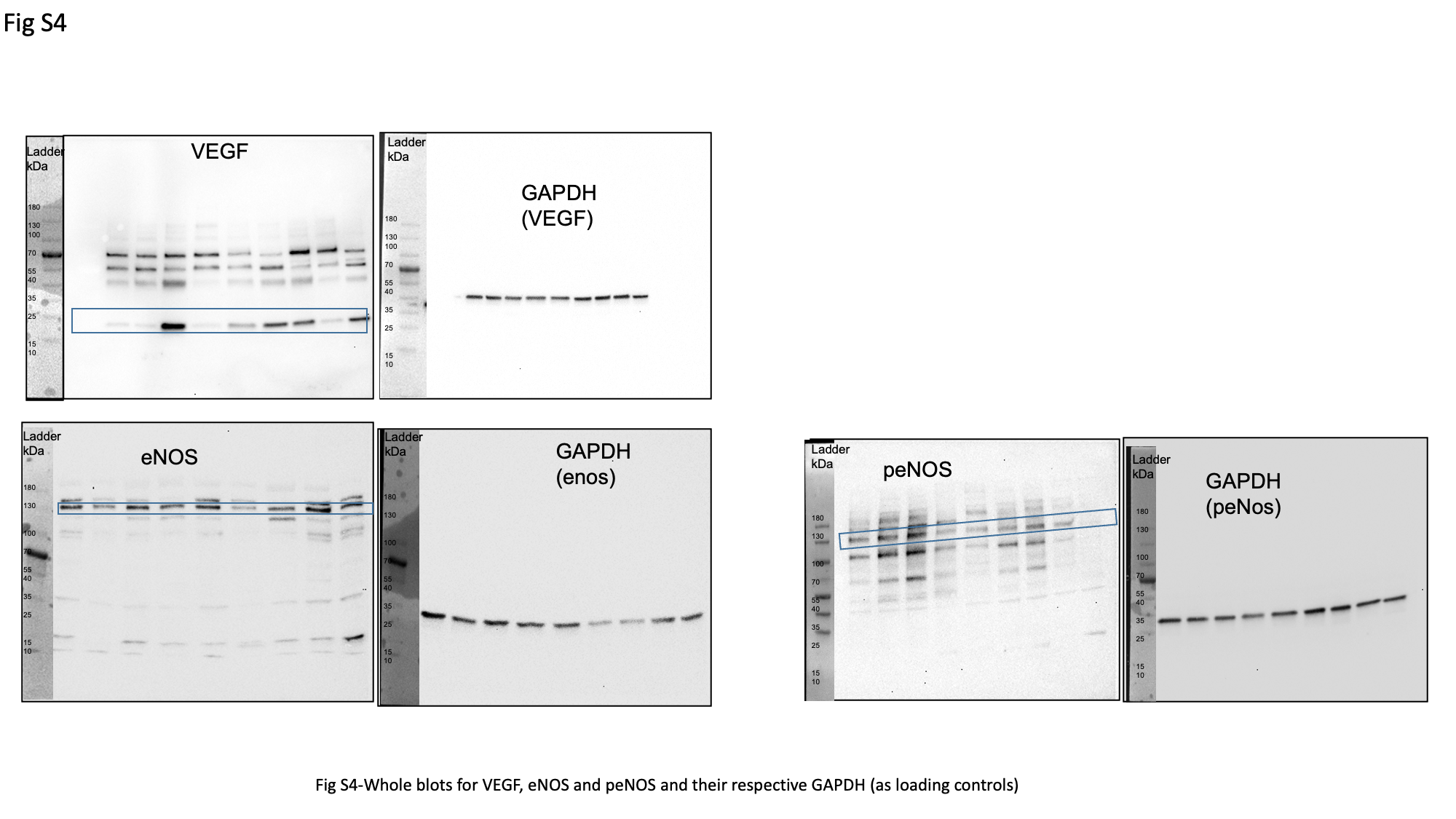

Supplement: Supplementary file 5 — Supplementary file5 (TIFF 6596 KB) [file 395_2025_1153_MOESM5_ESM.tiff]
